# Structure and luminescence of DNA-templated silver clusters

**DOI:** 10.1039/d0na01005g

**Published:** 2021-01-21

**Authors:** Anna Gonzàlez-Rosell, Cecilia Cerretani, Peter Mastracco, Tom Vosch, Stacy M. Copp

**Affiliations:** Department of Materials Science and Engineering, University of California Irvine California 92697-2585 USA stacy.copp@uci.edu; Nanoscience Center and Department of Chemistry, University of Copenhagen, Universitetsparken 5 2100 Copenhagen Denmark; Department of Physics and Astronomy, University of California Irvine California 92697-4575 USA

## Abstract

DNA serves as a versatile template for few-atom silver clusters and their organized self-assembly. These clusters possess unique structural and photophysical properties that are programmed into the DNA template sequence, resulting in a rich palette of fluorophores which hold promise as chemical and biomolecular sensors, biolabels, and nanophotonic elements. Here, we review recent advances in the fundamental understanding of DNA-templated silver clusters (Ag_*N*_-DNAs), including the role played by the silver-mediated DNA complexes which are synthetic precursors to Ag_*N*_-DNAs, structure–property relations of Ag_*N*_-DNAs, and the excited state dynamics leading to fluorescence in these clusters. We also summarize the current understanding of how DNA sequence selects the properties of Ag_*N*_-DNAs and how sequence can be harnessed for informed design and for ordered multi-cluster assembly. To catalyze future research, we end with a discussion of several opportunities and challenges, both fundamental and applied, for the Ag_*N*_-DNA research community. A comprehensive fundamental understanding of this class of metal cluster fluorophores can provide the basis for rational design and for advancement of their applications in fluorescence-based sensing, biosciences, nanophotonics, and catalysis.

## Abstract 2

DNA can stabilize silver clusters with sequence-tuned fluorescence. We review recent advances in understanding of the structures and properties of DNA-templated silver clusters, their rational design and ordered arrangement, and emerging challenges.

## Graphical abstract

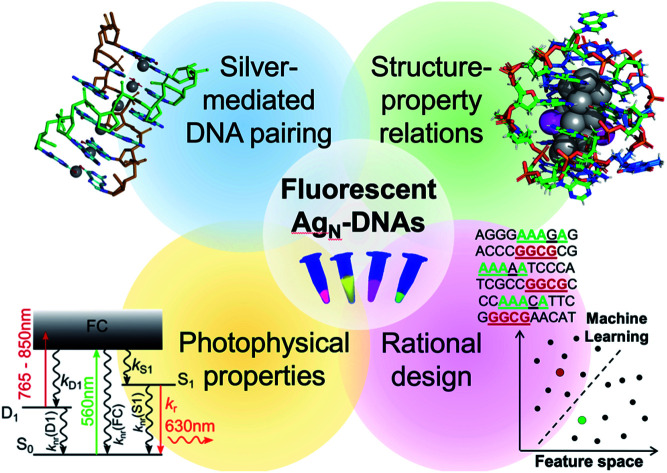

## 1. Introduction

Metal “nanoclusters” are the smallest of nanoparticles, consisting of only 2 to 10^2^ metal atoms and possessing remarkable properties which are very finely tuned by cluster size, shape, and charge. Bare metal clusters have been studied for decades in order to understand how single atoms with quantized energy levels transition into the continuous properties of bulk materials.^1^ Because the majority of unprotected metal clusters are unstable at ambient conditions, fundamental studies of metal clusters previously necessitated interrogation under ultra-high vacuum,^2^ which limited practical applications of these nanomaterials. This challenge has been overcome by the use of stabilizing ligands and supporting surfaces to bring metal clusters into the “real world” for applications such as catalysis, photonics, and electronics.^3^ In the past two decades, advances in synthetic chemistry have produced a “zoo” of different stable metal clusters passivated by molecular ligands, with cluster sizes that can even be tuned to atomic precision for especially fine control of their emergent properties.^4^ This review concerns an especially unusual type of ligand-stabilized metal cluster, the DNA-templated silver cluster (Ag_*N*_-DNA), which combines the atomic precision of cluster science with the programmability of DNA nanotechnology.

Ag_*N*_-DNAs are relatively new entrants into the diverse zoology of metal clusters, with unique properties that arise from their polynucleic acid ligands. Following work by the Dickson group on silver clusters stabilized in dendrimers^5^ and silver oxide films,^6^ in 2004, Petty, Dickson, and co-authors reported formation of fluorescent silver nanoclusters exhibiting 400–600 nm electronic transitions by chemically reducing an aqueous mixture of single-stranded cytosine-rich DNA and AgNO_3_.^7^ They then found that certain Ag_*N*_-DNAs exhibit very bright fluorescence^8^ and significant photostability and can be harnessed as biolabels.^9,10^ Gwinn, *et al.*, showed that the fluorescence colors of Ag_*N*_-DNAs depend sensitively on nucleobase sequence and that Ag_*N*_-DNAs prefer to form on single-stranded (ss) DNA rather than double-stranded (ds) DNA,^11^ motivating the important role played by silver–nucleobase interactions in Ag_*N*_-DNA formation. In the next few years, Ag_*N*_-DNAs were shown to be effective sensors for toxic metal ions,^12^ polynucleic acids,^13–15^ and other biomolecules.^16^ Together, these and other early studies generated considerable interest in harnessing DNA's sequence programmability for custom design of Ag_*N*_-DNA fluorophores tailored for precise sensing, fluorescence microscopy of cells and tissues, and direct integration into DNA nanotechnology schemes.^17–19^

**Fig. 1:**
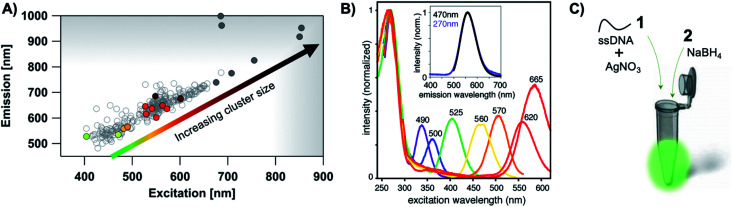
(A) The fluorescence colors of Ag_*N*_-DNAs, which are selected by DNA sequence, span a large spectral range from visible to NIR wavelengths and are correlated with cluster size.^20^ (B) Ag_*N*_-DNA excitation spectra exhibit a dominant peak in the visible to NIR spectral range as well as a UV excitation band corresponding exactly to the DNA template strand. Fluorescence spectra excited *via* the DNA bases (inset, purple) have the same lineshapes as spectra excited at the cluster's unique visible to NIR transition.^21^ Adapted from O'Neill, *et al.*, (ref. 21) with permission from the American Chemical Society. Copyright 2011. (C) Ag_*N*_-DNAs are chemically synthesized in aqueous solution by mixing ssDNA with a silver salt, followed by reduction with sodium borohydride.

The most remarkable characteristic of Ag_*N*_-DNAs is their sequence-dependent fluorescence. By employing DNA template strands with wide-ranging nucleobase sequences, a diverse color palette of Ag_*N*_-DNAs with fluorescence emission colors of 450 nm to 1000 nm has been developed^22,23^ (Fig. 1A), with quantum yields as high as 93% (ref. 24) and Stokes shifts as large as 5893 cm^−1^.^25^ Ag_*N*_-DNA fluorescence may be excited by at least two pathways, either directly at the cluster's size-, shape-, and charge-dependent excitation peak or universally *via* the DNA bases (Fig. 1B).^21,26^ Ag_*N*_-DNAs also exhibit unusual photophysics,^27^ intriguing dark states which can be harnessed for background-free fluorescence microscopy,^28–31^ light-up or color-switching behavior induced by various stimuli,^13,32–41^ and catalytic activity.^42,43^

Most well-studied ligand-stabilized metal clusters are protected by monolayers of small molecules such as thiolates^44^ and phosphines, with sizes smaller than or comparable to the metal clusters themselves.^45^ In contrast, Ag_*N*_-DNAs and their less-studied counterparts, Ag_*N*_-RNAs,^46^ are protected by bulky polynucleic acids much larger than the silver cluster. The structure and properties of these and other metal clusters stabilized by large macromolecular ligands, including proteins^47^ and dendrimers,^48^ are less understood than for monolayer-protected clusters, in part because bulky ligands can obscure resolution of cluster(s) and challenge crystallization, a necessary step for “solving” structure by X-ray crystallography. However, macromolecular ligands can also endow functionalities without the need for ligand exchange, adding a degree of versatility to applications of Ag_*N*_-DNAs and other macromolecule-stabilized nanoclusters.

Ag_*N*_-DNA synthesis is facile and is typically carried out by borohydride reduction of a solution of Ag^+^ and ssDNA in neutral pH aqueous solution (Fig. 1C). This method is robust to varying solution compositions, stoichiometries, and specific mixing/heating.^7,8,11,49–52^ In contrast to the simplicity of synthesis, achieving compositionally pure solutions of Ag_*N*_-DNAs is more challenging because reduction forms a heterogeneous mixture of silver-bearing DNA products containing varying numbers of silver atoms, *N*_tot_, and numbers of DNA strands, *n*_s_. The majority of these products are nonfluorescent^53^ and include clusters, Ag^+^–DNA complexes, and larger silver nanoparticles.^54,55^ It is also possible for a given DNA template to stabilize multiple different emissive cluster species,^56^ as has been observed for up to 25% of randomly selected DNA template sequences.^57^ Due to characterization of as-synthesized Ag_*N*_-DNAs without purification and/or due to fragmentation during mass spectrometry (MS), early reports underestimated Ag_*N*_-DNA sizes^8,11^ or found no correlation of fluorescence color with silver cluster size.^58^ A lack of awareness of this heterogeneity continues to hinder accurate characterization of Ag_*N*_-DNAs, and the assumption that the composition of Ag_*N*_-DNAs is uncorrelated to the optical properties of these nanoclusters still persists.^59^

The challenge of heterogeneity has been overcome by the use of reversed-phase high performance liquid chromatography (HPLC)^53,60^ and size-exclusion chromatography (SEC)^61,62^ to isolate a fluorescent Ag_*N*_-DNA of interest prior to compositional and spectral characterization. Additionally, development of gentle electrospray ionization (ESI) MS now enables compositional analysis without fragmentation of the Ag_*N*_-DNA product.^24,53,63^ Using tandem HPLC-MS with in-line UV/Vis and fluorescence spectroscopy, Schultz, *et al.* determined the compositions of several fluorescent Ag_*N*_-DNAs with fluorescence emission wavelengths, *λ*_em_, ranging from green to near infrared (NIR), finding that these clusters contained *N*_tot_ = 10-21 Ag atoms stabilized by *n*_s_ = 1–2 copies of the templating DNA strand.^53^ This ability to isolate and characterize compositionally pure solutions of Ag_*N*_-DNAs has enabled numerous future studies, leading to dramatic advances in our understanding of the structure–property relations of these nanoclusters, which we discuss in Section 3, and of their photophysical properties, which we discuss in Section 4.

This review focuses on the recent advances in fundamental understanding of Ag_*N*_-DNAs, with a particular emphasis on the recent detailed studies of compositionally pure Ag_*N*_-DNAs. We note that this review is timely because previous reviews which primarily focused on fundamental structure and properties^64–67^ are several years old and do not discuss recent breakthroughs, including the first reported Ag_*N*_-DNA crystal structures.^22,25,68,69^ Readers may also find a comprehensive list of DNA sequence/structure and optical properties for a large number of Ag_*N*_-DNAs by New, *et al.*,^70^ as well as previous reviews focused on the emerging applications of Ag_*N*_-DNAs as sensors and biolabels.^64,71–75^

Here, we summarize what is known about the connections among DNA sequence, Ag_*N*_ structure, and photophysical properties. We first review current understanding of the Ag^+^-mediated DNA base paired structures that are the synthetic precursors of Ag_*N*_-DNAs (Section 2). Next, we discuss current models for the structures of Ag_*N*_-DNAs, which have rapidly advanced due to detailed studies of compositionally pure Ag_*N*_-DNAs and a few breakthrough crystal structures (Section 3).^22,25,68,69^ Then we review current understanding of the excited state processes which lead to fluorescence in Ag_*N*_-DNAs and the unusual dark states exhibited by Ag_*N*_-DNAs (Section 4). We then discuss recent work to decode how DNA sequence selects Ag_*N*_-DNA properties by combining high-throughput experimentation and machine learning (Section 5). Finally, we review work on merging structural DNA nanotechnology with Ag_*N*_-DNAs for ordered arrangement of these nanoclusters (Section 6) and comment on opportunities and challenges facing the field of Ag_*N*_-DNA research (Section 7). It is our intent to provide a comprehensive and current picture of the properties of Ag_*N*_-DNAs which is accessible to researchers from many backgrounds, in order to aid others in developing applications of these unique nanoclusters and to inspire new experimental and computational studies of their fundamental properties.

## 2. Silver-mediated base pairing – precursors to Ag_*N*_-DNAs

A complete understanding of Ag_*N*_-DNA structure and sequence-dependent properties naturally begins with an understanding of Ag^+^–DNA complexation. This is because (i) Ag_*N*_-DNAs are formed by chemical reduction of Ag^+^–DNA complexes,^76^ (ii) high-resolution MS of HPLC purified Ag_*N*_-DNAs shows that usually about half of the silver atoms within Ag_*N*_-DNAs remain cationic,^24^ meaning that Ag^+^–DNA interactions play a key role in determining Ag_*N*_-DNA structure, and (iii) Ag^+^–DNA interactions are highly sequence-dependent,^54,55^ which may lead to the sequence dependence of Ag_*N*_-DNA size and fluorescence properties. Here, we review recent advances in fundamental understanding of Ag^+^–nucleobase interactions and secondary structures of Ag^+^–DNA complexes, with a focus on properties relevant to the formation and sequence-dependence of Ag_*N*_-DNAs. We note that this topic is a small part of the rich field of metal-mediated nucleobase pairing, an area of great interest as a route to expanded base–base interactions, DNA-based electronics, and sensing. We do not attempt to review this entire field here and point to excellent comprehensive reviews elsewhere on metal-mediated pairing of both natural and artificial bases^77–80^ and in the specific case of Ag and Au for natural DNA.^81^

### 2.1 Watson–Crick base pairing

**Fig. 2:**
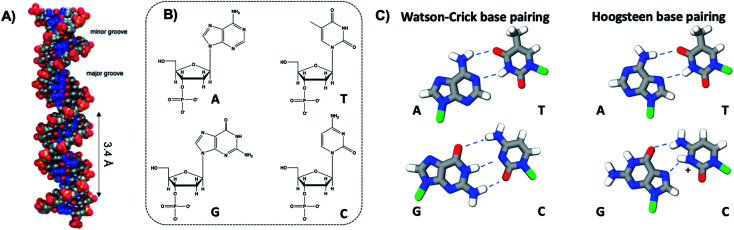
(A) Illustration of the double helix structure of natural Watson–Crick-paired B form DNA.^82^ Adapted from Bandy, *et al.* (ref. 82) with permission from the Royal Society of Chemistry. (B) Chemical structure of the natural nucleotides and (C) the H-bonded configurations of the nucleobases. Green regions represent the bonding positions of the nucleobases to the backbone.^83^

The four natural nucleobases of DNA are adenine (A), cytosine (C), guanine (G), and thymine (T). In canonical Watson–Crick (WC) pairing of dsDNA in B form, which is the most common structure of DNA *in vivo* (Fig. 2A), two complementary DNA strands join by hydrogen bonds (“H-bonds”) between A and T and between C and G, forming the familiar antiparallel double helix. C and G are held together by three H-bonds between the O2, N3 and N4 positions of C and the O6, N1 and N2 positions of G. In like manner, T and A are H-bonded through the O2 and N3 positions of T, and the N6 and N1 from A (Fig. 2C). This difference in the number of H-bonds between nucleobase pairs results in a weaker A–T WC bond as compared to C–G. The WC B-form double helix is further stabilized by hydrophobic stacking interactions between neighboring nucleobases. Additional less common DNA structures also exist, including WC paired A-DNA^84^ and Z-DNA^85^ and Hoogsteen base pairing.^86^ The extensive scientific understanding of DNA structure and thermodynamics has enabled the birth of DNA nanotechnology, which exploits DNA as a fundamental materials building block,^82^ engineering DNA sequence to achieve self-assembled predefined shapes,^18,87^ tuned colloidal interactions,^88–90^ and molecular computation.^91,92^

### 2.2 Ag^+^–nucleobase interactions of homobase strands

Silver cations (Ag^+^) are well-known to prefer binding to DNA nucleobases over the phosphate backbone at neutral pH.^93^ (Hg^2+^ possesses similar preference,^79,93–95^ but its significant toxicity prohibits applicability). This preference enables Ag^+^ intercalation into single base mismatches in WC-paired dsDNA, typically by interactions with nucleobase ring nitrogens.^93,96,97^ Cytosine (C) is especially well-known for affinity to Ag^+^, and this has been harnessed to expand the interactions among DNA oligomers, enabling Ag^+^-paired C–C mismatches,^96^ Ag^+^-folded i-motif secondary structures in C-rich DNA,^98^ Ag^+^-crosslinked DNA hydrogels,^99^ and DNA nanotubes.^100^ More recently, the study of Ag^+^-mediated nucleobase pairing has been extended to consider DNA that is unconstrained by WC base pairs. These studies show that silver can completely rearrange canonical DNA structures, as opposed to simply intercalating within base pair mismatches. Here, we review these recent advancements to provide context for the sequence-property connections that govern Ag_*N*_-DNAs (Section 5).

**Fig. 3:**
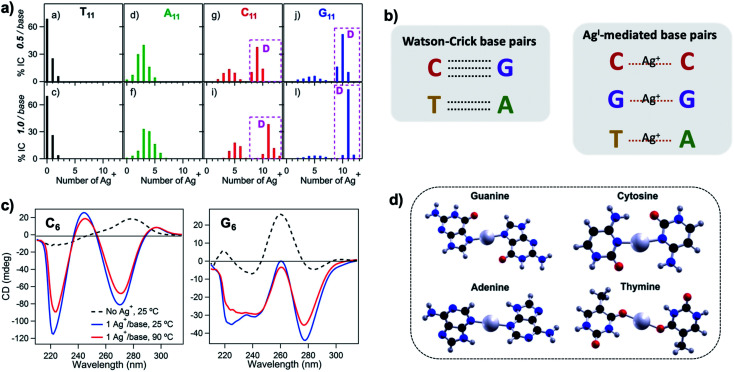
(A) Percentages of integrated counts (IC) for each product detected by ESI-MS for mixtures of Ag and 11-base homobase strands, at two different stoichiometries. Pink boxes and “D” represent Ag^+^-paired duplexes. (B) Summary of WC H-bonded base pairs and observed Ag^+^-mediated base pairs for duplexes of homobase strands. (C) Circular dichroism (CD) spectra for C_6_–Ag^+^–C_6_ and G_6_–Ag^+^–G_6_ show the significant thermal stabilities of these homobase-Ag^+^ duplexes. (D) Calculations of ground state geometries for Ag^+^-mediated homobase pairs finds planar geometries for G and C and nonplanar geometries for A and T, with binding energies of the trend G  >  C  >  A  >  T.^54^ (A, C and D) Adapted from Swasey, *et al.*, (ref. 54) with permission from Springer Nature. Copyright 2015.

To understand how Ag^+^ complexes with DNA in the case where the DNA does not form WC base pairs, Swasey, *et al.*, investigated interactions of Ag^+^ with homobase DNA strands.^54^ After solvent-exchanging DNA oligomers to remove any residual salts from oligomer synthesis, DNA was mixed with AgNO_3_ in an aqueous solution of ammonium acetate, followed by thermal annealing at 90 °C. Resulting products were analyzed by high-resolution negative ion mode ESI-MS to determine absolute composition by resolving the isotopic distribution (discussed in Section 3.1). Fig. 3A shows the compositions of all observed products for 11-base homobase strands. While C is best-known for affinity to Ag^+^ and was shown by Ritchie, *et al.*, to form Ag^+^-mediated duplexes,^52^ G was actually found to associate the greatest number of Ag^+^, with order of affinity: G > C > A > T. While the 4 types of natural nucleobases all formed Ag^+^-bearing single homobase strands, Ag^+^ also mediates formation of homobase duplexes for C and G. When two different single homobase strands are mixed, Ag^+^ only mediates the heteroduplex A–Ag^+^–T, completely replacing the WC A–T duplex. Ag^+^ also disrupts WC-paired C–G duplexes to instead form C–Ag^+^–C and G–Ag^+^–G homobase duplexes. Fig. 3B summarizes all observed pairing between homobase strands. C–Ag^+^–C and G–Ag^+^–G homoduplexes are remarkably stable, with C_6_–Ag^+^–C_6_ and G_6_–Ag^+^–G_6_ homoduplexes remaining intact at 90 °C, while C_6_–G_6_ WC duplexes melt below 20 °C (Fig. 3C).^54^

Quantum chemical calculations support greater stability of Ag^+^-mediated homoduplexes for C and G than for A and T. In the absence of steric factors, (base–Ag^+^–base)_N_ duplexes have higher bond energies than (base-Ag^+^)_N_ structures. Because C–Ag^+^–C and G–Ag^+^–G are nearly coplanar, with dihedral angles of 171.9° and 181.2° respectively, while T–Ag^+^–T and A–Ag^+^–A are nonplanar, with dihedral angles of 140° and 101.6°, respectively, C–Ag^+^–C and G–Ag^+^–G homoduplexes are expected to be significantly more stable (Fig. 3D). The A–Ag^+^–T bond is also non-coplanar, but its stability could be explained by the difference in size between A and T, which still allows adenine stacking interactions.^54^

The nucleobase sites with which Ag^+^ interacts differ from WC pairing. Simulations by the Lopez-Acevedo group have determined that pyrimidines C and T interact with Ag^+^ at the N3 position,^54,101^ which is deprotonated for thymine, while purines A and G coordinate with Ag^+^ at the N7 position.^54^ These binding sites correspond to the Hoogsteen region (Fig. 2C). However, these positions might change depending on the other nucleobase of the Ag^+^-bridging bond, as is the case for the C–Ag^+^–G bond, reported by Kondo, *et al.*, where the interaction with the purine base is through the N1 position, which is deprotonated.^102^

### 2.3 Ag^+^ mediates parallel strand orientation of highly stable homobase duplexes

**Fig. 4:**
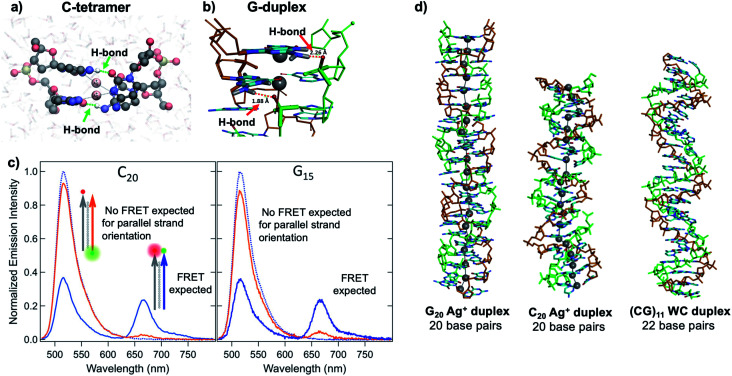
(A, B) DFT calculations predict the existence of novel inter-strand H-bonds in (A) C_2_–Ag^+^–C_2_ tetramers^101^ and (B) Ag^+^-paired G duplexes of varying lengths.^108^ These bonds add additional stability to Ag^+^-paired DNA duplexes. (A) Adapted from Espinosa Leal, *et al.*, (ref. 101) with permission from the American Chemical Society. Copyright 2015. (C) Emission spectra of Ag^+^-mediated C_20_ and G_15_ duplexes labeled with donor (green dot) and acceptor (red dot) dyes at 5′ end and 3′ end, respectively (orange curve) or with both dyes at 3′ ends (blue), compared to emission of the donor-bearing strand alone (blue dotted curve). Excitation is at 450 nm, which directly excites the donor only. Significant quenching of donor emission with concomitant acceptor emission (high FRET efficiency) clearly demonstrates that Ag^+^-mediated pairing of homo-duplexes arranges strands in a parallel orientation.^108^ (D) DFT-optimized structures of Ag^+^–DNA duplexes of G_20_ and C_20_ compared to WC duplexes of a mixed base (GC)_11_ show that Ag^+^ mediates formation of highly rigid duplexes of G homo-base strands and less rigid C homo-base duplexes. Ag^+^–DNA nanowires have parallel duplex strand orientation, as compared to canonical antiparallel strand orientation of WC duplexes.^108^ (B–D) Adapted from Swasey, *et al.*, (ref. 108) with permission from the American Chemical Society. Copyright 2018.

Quantum chemical and hybrid quantum mechanics/molecular mechanics (QM/MM) calculations by the Lopez-Acevedo group predicted that, unlike the antiparallel strand orientation of natural WC duplexes where one 5′-3′ strand pairs to a complementary 3′-5′ strand,^82^ C–Ag^+^–C duplexes and G–Ag^+^–G prefer a parallel orientation, with 5′ ends aligned.^101,103,104^ These helical duplexes align Ag^+^ along the helix axis and are stabilized not only by Ag^+^–nucleobase interactions but also by novel interplanar H-bonds (Fig. 4A and B).^101,103,104^ Calculated electronic CD spectra of C_2_–Ag^+^–C_2_ tetramers agree well with experimentally measured CD spectra, further supporting a parallel arrangement.^101^ However, other experimental studies report varying behavior. One study of the conductivity of C–Ag^+^–C duplexes achieves antiparallel duplex formation of strands confined at ends to a metal surface and scanning probe tip.^105^ As discussed in Section 2.4, both parallel^106^ and antiparallel structures are reported^102,107^ for mixed base strands.

Recent study of unconstrained homobase strands confirms parallel duplex structure for C–Ag^+^–C and G–Ag^+^–G by utilizing Förster Resonance Energy Transfer (FRET) experiments to determine DNA strand orientation and ion mobility spectrometry (IMS) MS coupled with density functional theory (DFT) calculations to elucidate structure.^108^ Variations in FRET efficiency between donor and acceptor dyes coupled to ends of two DNA strands support parallel Ag^+^-paired C homobase duplexes and G homobase duplexes (Fig. 4C). This parallel orientation was further demonstrated by IMS-MS experiments coupled with DFT calculations of collision cross sections (CCS), which support high aspect ratios for both guanine and cytosine duplexes, consistent with rigid, wire-like structures (Fig. 4D). Based on CCS values and their agreement to calculated values, the G–Ag^+^–G duplex is found to be more rigid because nucleobases form additional H-bonds with the phosphate groups in the backbone, whereas the C–Ag^+^–C duplex lacks these extra bonds and is more flexible.

### 2.4 Ag^+^–nucleobase interactions of mixed base strands

**Fig. 5:**
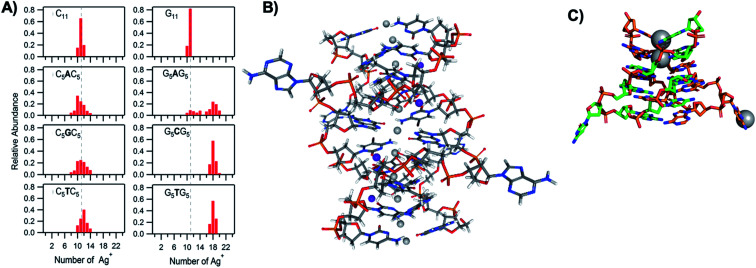
(A) MS-determined distributions of the numbers of Ag^+^ attached to DNA oligomers (sequences indicated on each graph) determined by relative integrated intensity of individual mass peaks relative to all silver-bearing duplexes. Single-base mutations in G-rich oligomers enable attachment of many more Ag^+^.^55^ Adapted with permission from Swasey, *et al.* (ref. 55) with permission from the Institute of Physics. (B) Crystal structure of an Ag^+^-paired DNA duplex with antiparallel orientation. End-to-end assembly of these duplexes forms uninterrupted nanowires. Protruding adenines foster assembly of multiple wires into 3D lattices.^102^ Silver atoms are shown in gray and potassium atoms in purple. Image created from PDB ID 5IX7 with NGL Viewer.^112^ (C) Structure of a dimer of 5′-GCACGCGC-3′ (orange, green) paired by two Ag^+^ (grey). The third Ag^+^ (bottom right of structure) supports supramolecular assembly of the structure during crystallization.^106^ Image created from PDB ID 5XJZ with PyMOL.

The vast majority of reported Ag_*N*_-DNA nanoclusters are stabilized by DNA strands with mixed base sequences. To understand how heterobase strands recruit Ag^+^, Swasey and Gwinn examined ten noncomplementary 11-base DNA strands, determining composition of Ag^+^–DNA complexes by ESI-MS (HPLC-MS was employed to analyze very heterogenous samples).^55^ Interestingly, strands with sequences formed by single-base “mutations” of C_11_ increase the distribution of the number of Ag^+^ attached to duplexes, and inclusion of mutations in G_11_ homobase strands can significantly increase the average number of Ag^+^ by up to 7 or 8 Ag^+^ per duplex (Fig. 5A). Both homobase and heterobase Ag^+^-mediated duplexes were found to be stable in various solution conditions, significant Mg^2+^ concentrations, and high concentrations of urea (a strong denaturant). While the chemical structures adopted by these heterobase duplexes are not known, the differences in Ag^+^ recruitment have important implications for the origins of Ag_*N*_-DNA sequence dependence, which we discuss in Section 5.

Kondo, *et al.*, recently developed remarkable uninterrupted Ag^+^–DNA “nanowires” and solved their 3D structure, determining formation of consecutive Ag^+^-paired duplexes with antiparallel orientation.^102^ The DNA strand used to form the Ag^+^ wires, GGACT(^Br^C)GACTCC, is a near-complement which forms a WC-paired homodimer with one C–C mismatch at room temperature in biologically relevant salt concentrations (determined using UNAfold software^109,110^). C–Ag^+^–C, G–Ag^+^–G, T–Ag^+^–T, and C–Ag^+^–G bonds were observed in the nanowire, and interestingly, not all nucleobases in the strand participate in the principal linkage between strands. A's protrude outwards (Fig. 5B) and contribute to crystal-packing through formation of AT–Ag^+^–A triplets and AA stacking interactions. Thanks to the near reversibility of the sequence used, and because A's do not participate in duplex bonding, most nucleobases are bonded to a like base in the partner strand, with only two C–Ag^+^–G pairs observed. Pairing between the two strands occurs with a one-position shift, enabling formation of nanowires up to 0.1 mm long. Despite the antiparallel orientation and the C–Ag^+^–G pairs, in which the G is bonded through the N1 position, the system clearly does not obey WC pairing because the main interaction sites lie in the Hoogsteen region. Furthermore, the propeller twist angles obtained are larger than in WC pairing, which can be explained by repulsions between amino and carbonyl groups of opposite bases.^102^

Liu, *et al.* solved the 3D structure of another Ag^+^-paired mixed base strand, 5′-GCACGCGC-3′, which forms curved dimers attached by one G–Ag^+^–G bond and one C–Ag^+^–C bond, with parallel strand orientation (Fig. 5C).^106^ In this structure, it is only like bases which participate in Ag^+^-mediated pairing, and these base pairs are less planar (Fig. 5C) than the nearly coplanar angles predicted by previous DFT calculations.^54^ This suggests that mixed base strands can accommodate a wide range of Ag^+^-mediated base interactions beyond just linear wires. This 8-base sequence was also uncovered in an unrelated study using machine learning methods to design templates for Ag_*N*_-DNAs with fluorescence emission in the 600 nm < *λ*_em_ < 660 nm window.^111^ This surprising coincidence suggests that some Ag_*N*_-DNAs are formed by chemical reduction of nontrivial Ag^+^–DNA complexes.

Very recently, the Kohler group reported evidence for a parallel oriented Ag^+^-mediated duplex of C_20_ with significant “propeller” twist of the C–Ag^+^–C base pairs, as has been reported in the studies above. This evidence was based on strong agreement between experimentally measured and calculated CD spectra.^113^ The authors note that such twisting has been associated with reduced flexibility of DNA,^114^ and this enhanced rigidity agrees with the past IMS studies of C–Ag^+^–C duplexes described in Section 2.3.^108^

### 2.5 Relevance of Ag^+^-mediated base pairing for Ag_*N*_-DNAs

As synthetic precursors of Ag_*N*_-DNAs,^76,115^ Ag^+^–DNA complexes are the scaffolds that reorganize into the cluster-stabilizing cage of an Ag_*N*_ and, at least in part, provide the Ag^+^ “fuel” to grow the Ag_*N*_ upon reduction. Early studies which found that Ag_*N*_-DNA do not form on completely dsDNA templates^11^ have led to the false assumption that the Ag_*N*_ can always be confined within single-stranded regions of WC-paired DNA structures such as hairpins^116,117^ or other dsDNA structures with ssDNA regions,^118^ based on the assumption that WC DNA secondary structure is preserved in the presence of Ag^+^. The dramatic rearrangement of DNA homobase and heterobase strands by Ag^+^, together with the significant thermal and chemical stabilities of Ag^+^-mediated DNA duplexes,^54,55^ call into question whether this assumption is accurate. It is more likely that Ag^+^ can invade and unravel WC dsDNA under appropriate conditions, rearranging secondary and tertiary structures which then further evolve upon chemical reduction. This has been suggested by several careful studies,^49,119–121^ and Ag^+^ has also been shown to rearrange the well-known G-quadruplex structure^108^ and i-motif structure.^113^ Further studies will be needed to determine to what degree DNA secondary structure is preserved after Ag_*N*_-DNA synthesis, especially when Ag_*N*_-DNAs are incorporated into the larger DNA structures discussed in Section 6.

## 3. Structure–property relations – discerning the geometries of Ag_*N*_-DNAs

The past several years have seen dramatic improvement in our understanding of Ag_*N*_-DNA chemical structures and their relation to optical properties, culminating in reports of the first crystal structures of Ag_*N*_-DNAs.^22,25,68,69^ Nearly all of these advancements have been enabled by compositionally pure Ag_*N*_-DNAs isolated using HPLC^53^ or SEC.^37,38,62^ These techniques separate different DNA complexes by exploiting variations in size and polarity that are induced by different silver products on the DNA template strands. (Methods for isolating Ag_*N*_-DNAs using HPLC have been reviewed in detail previously.^66^) Purification prior to characterization is crucial because as-synthesized solutions contain multiple dark and fluorescent products, including Ag nanoparticles, Ag_*N*_-DNAs and Ag^+^–DNA complexes, as supported by LC-tandem MS.^24,57^ Even though one would naively expect Ag_*N*_-DNA properties to be similar in the as-synthesized and purified states, a recent report by Gambucci, *et al.*, showed different rotational correlation times, indicating that synthesis fragments could be attached to the Ag_*N*_-DNAs, *e.g.* by Ag^+^-mediated interactions.^122^ Compositional analysis methods that only infer average stoichiometry of the entire heterogeneous as-synthesized solutions may misjudge the number of silver atoms within an Ag_*N*_-DNA and cannot resolve the number of DNA strands *n*_s_ that stabilize a single cluster, and MS performed directly on as-synthesized samples makes it challenging to identify the fluorescent Ag_*N*_-DNA of interest from the other products formed during synthesis. Here, we primarily review structural studies of HPLC-isolated Ag_*N*_-DNAs with bright visible or NIR fluorescence, which have thus far been found to contain *N*_tot_ = 10–30 Ag atoms,^23,24,53,57,123^ as opposed to earlier reports of dimers or trimers of Ag.^8,11^ We then discuss other studies that focus on inference of the conformation of the DNA template strand(s) around the Ag_*N*_. Unless indicated, all Ag_*N*_-DNAs discussed are compositionally pure.

### 3.1 Mass spectrometry to determine Ag_*N*_-DNA composition

Prior to the breakthrough crystallographic structures of Ag_*N*_-DNAs solved in 2019,^22,69^ efforts to discern Ag_*N*_-DNA structure mainly employed correlations of experimentally measured absorption, excitation, and/or emission for Ag_*N*_-DNAs of known composition with computational studies or simple models. These past studies do not provide the same level of structural detail as the recent crystal structures but do provide a more comprehensive picture of the structure–property relations of Ag_*N*_-DNAs in general, with detailed studies on about 20 different HPLC-purified Ag_*N*_-DNAs as compared to the smaller number of crystal structures currently available.^22,25,68,69^

Since metal cluster size, charge, and geometry strongly determine properties, accurate characterization of composition is a key step towards building a fundamental understanding of metal clusters.^45^ It is well-established that other ligand-stabilized metal clusters are only partially reduced because a fraction of the metal atoms in the cluster are bound to the surrounding ligands and that the number of remaining effective valence electrons in the cluster core is a major determinant of the electronic properties of the cluster.^124–127^ Partially oxidized Ag_*N*_-DNAs were proposed by Ritchie, *et al.*, based on the oxygen and chloride dependence of the fluorescence of C_12_-stabilized clusters.^128^ An experimental method to not only count the numbers of silver atoms *N*_tot_ and DNA strands *n*_s_ in a purified sample of Ag_*N*_-DNA but also to separate *N*_tot_ into neutral (*N*_0_) and cationic (*N*_+_) silver content can yield insights into how Ag_*N*_ clusters are ligated to the DNA and enable computational studies of their electronic properties.^126,127,129^ High resolution mass spectrometry (HR-MS) is an ideal tool to achieve this goal because HR-MS can be used to determine both ion mass, *M*, and charge, *Z*, rather than just the ratio *M*/*Z*, by resolving the isotope pattern that arises due to natural variation in isotopic abundances of elements. Koszinowski and Ballweg determined the charge of an Ag_6_^4+^-DNA by comparing the experimentally measured isotope pattern to the calculated distribution of this cluster.^63^ To characterize the properties of fluorescent Ag_*N*_-DNAs, this approach has been developed in conjunction with chromatographic purification by the Gwinn and Petty groups.^24,130^

**Fig. 6:**
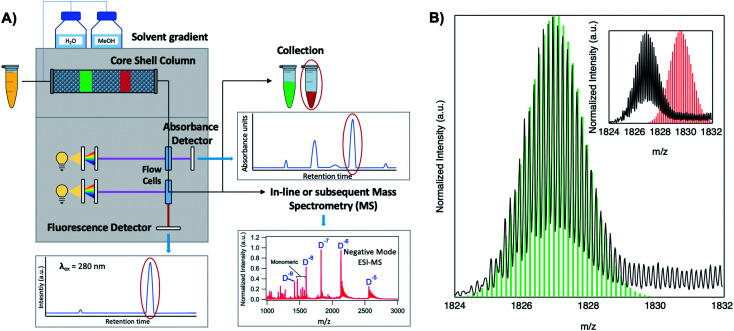
Tutorial schematic of tandem HPLC-MS with in-line UV/Vis and fluorescence spectroscopy, developed by Schultz and Gwinn.^53^ (A) In this illustration, the initial sample (yellow tube) is a mixture of products including multiple dark Ag-DNA complexes, one green-fluorescent Ag_*N*_-DNA species, and one red-fluorescent Ag_*N*_-DNA species. The as-synthesized Ag_*N*_-DNA solution is injected into an HPLC outfitted with a core–shell C_18_ column for reverse-phase, ion-pair (IP) HPLC. Products are separated due to slight variations in column affinity with a water–methanol gradient and a triethyl ammonium acetate (TEAA) IP agent. By monitoring both absorbance at ∼260 nm, which correlates to the absorbance of DNA, and fluorescence emission (*e.g.* UV-excited fluorescence^21^), correlation of absorbance and fluorescence chromatogram peaks indicate elution of a fluorescent Ag_*N*_-DNA species. We note that the chromatogram schematics are simplified for illustration; real chromatograms are more complex.^53^ Products of interest can either be sized by in-line negative-ion mode ESI-MS or collected for subsequent ESI-MS. A mass spectrum for a previously studied 30-atom NIR-emissive product is shown in the bottom right.^23^ Both monomeric and dimeric (labeled “D”) products are visible, with spacing of the isotopic peaks indicating the charge state of each product (labeled as superscript of “D”) for dimeric products. (B) Experimental mass spectrum of the Ag_30_-DNA product at the 7− charge state dimeric product (labeled D^−7^ in (A)) is shown in black, with the calculated mass distribution (green bars) for a product with 2 DNA strands, *N*_0_ = 12 Ag^0^, and *N*_+_ = 18 Ag^+^.^23^ Inset: compares the experimental spectrum^23^ with the calculated distribution for a product with no charged silvers (2 DNA strands and 30 Ag^0^), illustrating how the shift between the experimental and calculated isotopic finger distribution can be used to accurately determine the numbers of Ag^0^ and Ag^+^ in an Ag_*N*_-DNA product. Mass spectra are adapted from Swasey, *et al.*, (ref. 23) with permission from the Royal Society of Chemistry.

Because DNA is easily deprotonated, negative ion mode ESI-MS is suitable to resolve weakly bound, noncovalent DNA complexes^131,132^ and has been used to size a variety of silver-bearing DNA complexes.^53–55,57,60,63,108^ (While more sensitive, positive mode ESI-MS can oxidize encapsulated clusters during electrospray, hindering full determination of composition.^133^) The mass spectrum of an Ag_*N*_-DNA product may be collected either by tandem HPLC-MS (Fig. 6A) or by direct injection into the MS following previous purification. Determination of *N*_0_ and *N*_+_ for an Ag_*N*_-DNA from its mass spectrum is illustrated in Fig. 6. First, the charge state *Z*- of a *M*/*Z* (mass to charge ratio) peak is determined by the spacing between adjacent peaks of the isotope pattern: these peaks are spaced by 1/*Z* (*Z* is defined as a positive integer). For example, Fig. 6B shows the 7- charge state (*Z* = 7, minus sign is due to negative ion mode MS) of an Ag_30_-DNA, with individual isotope peaks separated by 1/7.^23^ (The product shown in Fig. 6B has a charge of -7*e*, where *e* is the fundamental unit of charge). The total charge of the complex corresponding to this *M*/*Z* peak is equal to the charge of the number of silver cations, *eN*_+_, minus the charge of the number of protons removed from the DNA, *en*_pr_, to reach the total charge of −*eZ*:

1 −*eZ* = *eN*_+_ − *en*_pr_

Note that as number of silver cations, *N*_+_, increases, more protons must be removed from the complex to reach charge state *Z*. Then, because *n*_pr_ protons have been removed from the Ag_*N*_-DNA complex, the measured total mass *M* (in amu) is:

2 *M* = *m*_DNA_*n*_s_ + *m*_Ag_ (*N*_+_ + *N*_0_) − *n*_pr_

where *m*_DNA_ is the DNA template strand mass, *n*_s_ is the number of DNA strands in the complex, and *m*_Ag_ is the silver atom mass (the mass of a proton is treated here as 1 amu). In the case of well-resolved patterns, *N*_+_ and *N*_0_ may be determined by calculating the isotope distribution pattern for varying values of *N*_+_, and thus *n*_pr_, to determine the charge which best matches the isotope pattern (Fig. 6B).^24^ If signal is too low to precisely resolve the isotope pattern, charge may be inferred by comparing Gaussian fits of the calculated and experimentally measured isotope patterns.^55^ Using this method, Schultz, *et al.*, determined that approximately half of the silver atoms within Ag_*N*_-DNA are cationic in nature.^24^

HR-MS is advantageous for determination of *n*_s_, *N*_0_, and *N*_+_ without ambiguity, provided that gentle enough ESI is applied. Inductively coupled plasma-atomic emission spectroscopy (ICP-AES) has also been used to determine the composition of purified Ag_*N*_-DNAs,^37^ although *n*_s_ cannot be determined by this method. This has led to underestimates of the sizes of Ag_*N*_-DNAs with *n*_s_ > 1, which were later characterized by HR-MS.^24^

### 3.2 Experimental evidence for elongated cluster geometry

The first experimental evidence that Ag_*N*_-DNA cluster geometry differs from globular (or quasi-spherical) arose by comparing the absorption spectra of compositionally pure Ag_*N*_-DNAs (whose *N*_+_ and *N*_0_ were determined by HPLC-MS) to the experimental and computed spectra of bare Ag_*N*_ in the gas phase which have similar numbers of effective valence electrons (equal to *N*_0_). The electronic properties of ligand-stabilized metal clusters depend on the number of effective valence electrons in the cluster core, not only the total number of atoms *N*_tot_, and these valence electrons can delocalize to form “superatomic” orbitals.^125^ Thus, it is most appropriate to compare the properties of Ag_*N*_-DNAs with bare silver clusters having like numbers of effective valence electrons. Due to ligation with the nucleobases,^24^ not all Ag atoms in an Ag_*N*_-DNA will contribute to the valence electron count. To determine the effective valence electron count of an Ag_*N*_-DNA, we subtract the charge of the cluster, *N*_+_, from the total number of atoms in the cluster, *N*_tot_, finding that the number of effective valence electrons is *N*_0_ = *N*_tot_ – *N*_+_.

**Fig. 7:**
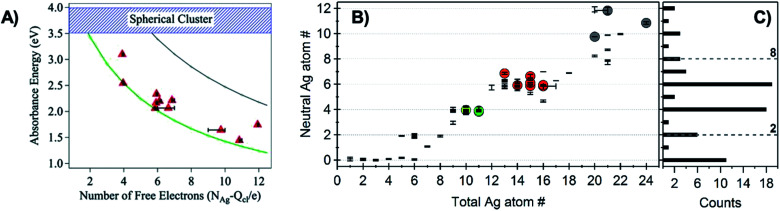
(A) Peak absorbance energies for purified Ag_*N*_-DNAs characterized by MS as a function of the number of effective free electrons in the cluster (red).^24^ Experimental data are better described by simulations of silver nanorods with 1-atom cross-sections (green line) than for thicker nanorods with 6-atom cross-sections (gray line) or spherical clusters (blue band).^136^ Adapted from Schultz, *et al.*, (ref. 24) with permission from John Wiley and Sons. Copyright 2013. (B) Neutral Ag atom numbers, *N*_0_, as determined by HR-MS for HPLC-purifiable Ag_*N*_-DNAs, including brightly fluorescent Ag_*N*_-DNAs (colored dots with RGB color matching fluorescence wavelength (NIR = gray)) and Ag_*N*_-DNAs without measurable fluorescence (black). (C) Histogram of *N*_0_ values show abundances of clusters with even *N*_0_ as compared to magic numbers 2 and 8 predicted by the spherical “superatom” model.^57^ (B and C) Adapted from Copp, *et al.*, (ref. 57) with permission from the American Chemical Society. Copyright 2014.

Schultz, *et al.*, found that the numbers and locations of peaks in the optical spectra of Ag_*N*_-DNAs differ markedly from their globular bare cluster counterparts. Naked Ag_*N*_ with cluster sizes *N* = 2 to 20 exhibit globular geometries and absorption spectra with multiple UV transitions in the 3 to 5 eV spectral range.^134,135^ In contrast, purified Ag_*N*_-DNAs have much simpler spectra with single dominant peaks in the visible to NIR range <3 eV, whose locations strongly depend on *N*_0_,^24,53^ and an additional UV absorption band due to the DNA ligand (Fig. 1B).^21^ The energies of the visible to NIR absorbance peaks of Ag_*N*_-DNAs with varying *N*_0_ can be described by quantum chemical calculations by Guidez and Aikens for linear atomic chains of silver (Fig. 7A).^136^ Based on these results and on the significant degree to which Ag_*N*_-DNA emission is polarized, as observed by spectroscopy of single Ag_*N*_-DNAs, a rod-like structure for Ag_*N*_-DNAs was proposed by the Gwinn group.^24^ Following this model, Ramazanov and Kononov used DFT-calculated electronic excitation spectra to argue that thread-like clusters show better agreement with experimental data than planar clusters.^137^

A rod-like geometry is also supported by the magic *N*_0_ numbers of Ag_*N*_-DNAs. The energetic stability of many ligand-stabilized metal clusters can be described by the “superatom” model, which states that the effective valence electrons in the cluster core are characterized by an electronic shell structure, similar to the shell structure of the atomic nuclei.^124,125^ For spherical metal clusters, closed shells are expected for *N*_0_ = 2, 8, …, resulting in enhanced abundances of clusters of these sizes due to their significantly enhanced stabilities (the same behavior is observed for gas phase bare metal clusters^2^). Copp, *et al.*, performed a large-scale study of Ag_*N*_-DNAs stabilized by ∼700 different DNA templates, finding enhanced abundances of Ag_*N*_-DNAs with even numbers of neutral silver atoms: green-emissive Ag_*N*_-DNAs with *N*_0_ = 4, red-emissive Ag_*N*_-DNAs with *N*_0_ = 6, and larger NIR-emissive *N*_0_ = 10–12 Ag_*N*_-DNAs (Fig. 7B); the spherical magic numbers of 2 and 8 were not especially abundant (Fig. 7C). This behavior is consistent with clusters that are significantly aspherical, for which additional energy stability is primarily conferred by pairing of electron spins, resulting in enhanced stabilities for even values of *N*_0_.^57^

Chiroptical properties of Ag_*N*_-DNAs have been well-modeled by a thread-like cluster structure. Because circular dichroism (CD) spectroscopy is extremely sensitive to specific geometrical structure and can be calculated using first-principles methods, CD allows a direct interface with theory. Swasey, *et al.*, measured the CD spectra of four Ag_*N*_-DNAs spanning the visible to NIR color palette. Quantum chemical calculations for bare atomic Ag chains with a chiral twist agree well with the experimental spectra.^138,139^ Similarity between CD spectra of Ag_*N*_-DNAs and their unreduced Ag^+^–DNA precursors was also observed, pointing to the role played by the Ag^+^–DNA complex in dictating final cluster structure^138^ (we note that recent studies suggest the Ag_*N*_ itself is not the cause of the CD signal observed for Ag_*N*_-DNA but that the DNA–silver interaction of the intrinsically chiral DNA plays a crucial role in generating chiroptical properties of these clusters^127^).

Past studies have found that classical theories which describe collective electronic excitations of colloids,^140^ such as Mie-Gans theory,^141,142^ show surprising agreement with the optical properties of small metal clusters,^134,143^ particularly for longitudinal plasmonic modes.^144^ Copp, *et al.*, examined whether Ag_*N*_-DNAs can also be described by classical models, applying Mie-Gans theory to HPLC-purified Ag_*N*_–DNAs with 400–850 nm cluster excitation wavelengths and numbers of effective valence electrons, *N*_0_, determined by HR-MS in order to elucidate the aspect ratios of these clusters. Application of Mie-Gans theory to this experimental data predicted prolate cluster geometry, with aspect ratios of 1.5 for *N*_0_ = 4 up to ∼5 for *N*_0_ = 12. (The currently reported crystallographic structures for Ag_*N*_-DNAs do not yet have determined charges,^22,25,68,69^ so these aspect ratios remain unconfirmed by solved structures.) Ag_*N*_-DNAs with *N*_0_ ≥ 6 displayed shifts in peak excitation wavelength dependent on solvent dielectric, as is expected for a collective electronic excitation^145^ and observed for larger metal nanoparticles;^146^ such sensitivity may be useful for applications. The increase in peak excitation wavelength and extinction coefficient with increasing cluster core size *N*_0_ (ref. 147) is a characteristic shared by the longitudinal collective electronic excitation sustained by rod-like metal clusters^136,144,148^ and larger metal nanoparticles.^145,149,150^ While the proper definition of a plasmon *versus* a collective electronic excitation at the cluster scale remains debated, other molecular-scale systems have also been shown to exhibit plasmon-like behavior.^151–154^ The Sánchez group simulated toy model Ag_*N*_-DNAs with magic number sizes,^57^ finding that a neutral silver cluster rod surrounded by nucleobase-bound Ag^+^ is generated when a partial charge is placed on the cluster. When excited, these clusters supported longitudinal plasmon-like modes.^129^ Intriguingly, single Ag_*N*_-DNAs studied at temperatures below 2 K exhibit surprisingly broad spectral linewidths.^155^ For larger nanoparticles, surface plasmon resonance broadening is understood to arise from dephasing processes for multiple delocalized electrons,^156^ but such effects are less well understood at the cluster scale. As silver cluster rods, Ag_*N*_-DNAs may provide a unique platform to investigate these important questions. It remains to be determined whether the optical transitions in Ag_*N*_-DNAs are collective or plasmonic-like, and further experimental and theoretical studies are needed to reveal which models are most suitable to represent the behavior of Ag_*N*_-DNAs.

### 3.3 X-ray and IR spectroscopy of solution-state Ag_*N*_-DNAs

**Fig. 8:**
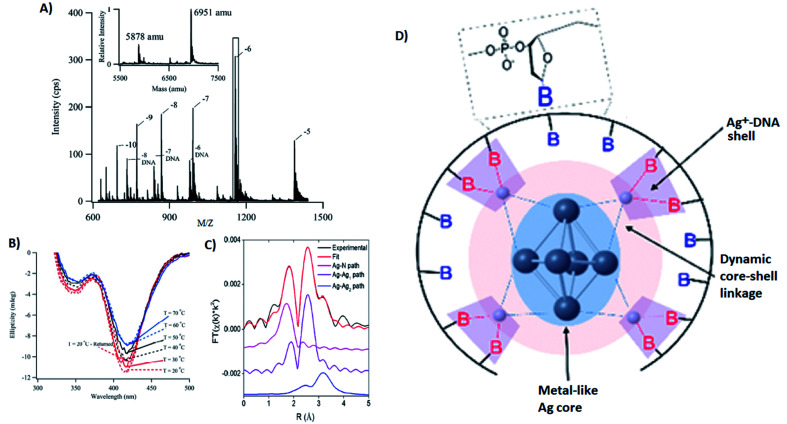
(A) Mass spectrum of 20-base DNA-templated Ag_*N*_. The peaks labeled −10 to −5 correspond to the ions of the Ag_*N*_-DNA, while the peaks labeled −8 DNA to −6 DNA are ascribed to the ions of the bare DNA strand. The inset shows the zero-charge spectrum that identifies the native DNA at 5878 amu and the DNA with 10 Ag at 6951 amu. (B) CD spectra of Ag_*N*_-DNAs at different temperatures. (C) Ag K-edge EXAFS trace of the solution state Ag_*N*_-DNAs. The experimental data (black) was fitted (red) with three individual scattering paths (magenta, purple and blue) displayed separately. (D) Suggested Ag_*N*_-DNA structure after combining all information from MS and EXAFS measurements.^130^ Adapted from Petty, *et al.*, (ref. 130) with permission from the American Chemical Society. Copyright 2016.

Several groups have applied X-ray spectroscopy, nuclear magnetic resonance (NMR), and infrared (IR) spectroscopy to probe the structures and silver–DNA interaction in purified Ag_*N*_-DNAs. To interrogate stoichiometry, oxidation state, ligand environment, and structure of a violet-absorbing Ag_*N*_-DNA, Petty, *et al.* used ESI-MS, X-ray absorption near edge structure (XANES), and Extended X-ray Absorption Fine Structure (EXAFS).^130^ This dimly fluorescent cluster has absorbance peaked at 400 nm but converts into a NIR-emissive species upon perturbation of its DNA template strand.^33^ MS data (Fig. 8A) and Ag L_3_ edge XANES spectra establish the SEC-purified violet cluster to be an Ag_10_^6+^. CD spectra of this Ag_10_^6+^ remain stable above 70 °C, pointing to the temperature stability of the DNA–Ag interaction (Fig. 8B). Ag K-edge EXAFS was used to probe organization of Ag atoms and Ag–nucleobase interactions. The experimental EXAFS trace (black) was fitted to three individual scattering paths (Fig. 8C) to infer specific bond lengths and coordination numbers. Based on these results, the authors proposed an octahedral cluster structure (Fig. 8D). While creating an accurate model from EXAFS data is nontrivial given the vast number of possible geometries in such a complicated system, the model contains several structural elements later found in the crystal structure of an Ag_16_ investigated by Cerretani, *et al.*,^69^ including an octahedral structural motif, limited to no classical WC pair interactions, and the fact that not all nucleobases interact with the Ag_*N*_.

Petty, *et al.*, later developed a different Ag_10_^6+^ stabilized by an altered DNA template which forms a hairpin at one terminus.^157^ This altered cluster has the same oxidation state as the “violet” cluster above and can be reversibly converted by manipulation of the hairpin region. The altered cluster is highly fluorescent and has red-shifted absorbance. Using differences in EXAFS data between the two clusters, the altered cluster is proposed to have a more extended and distinct metal-like core, presumably due to variations in coordination with the DNA ligand. These variations are supported by later studies using activated electron photodetachment MS.^121^

**Fig. 9:**
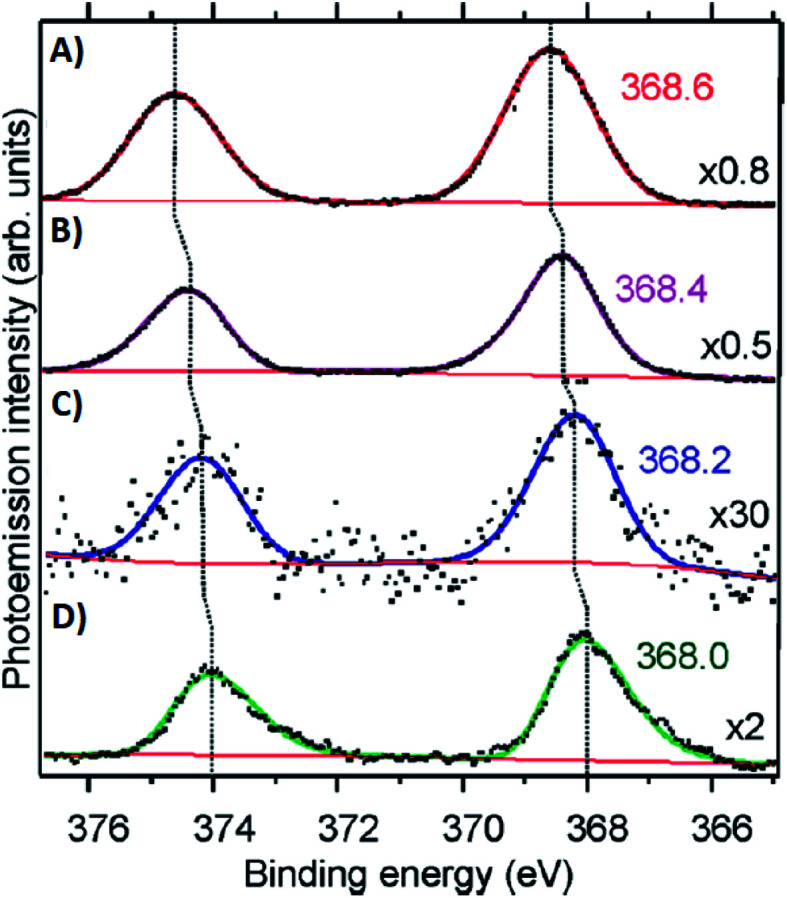
Ag 3d peak doublet for (A) Ag^+^–DNA complexes, (B) AgNO_3_ salt, (C) HPLC-purified fluorescent Ag_*N*_-DNAs (which combine both neutral and cationic Ag) and (D) metallic Ag nanoparticles. Adapted from Volkov, *et al.*, (ref. 158) with permission from the American Chemical Society. Copyright 2017.

Volkov and co-workers used X-ray photoelectron spectroscopy (XPS) to study an HPLC-purified Ag_*N*_-DNA.^158^ The oxygen spectra are similar with and without Ag^+^, supporting that Ag^+^ prefers to bind to nitrogen when no reducing agent has been added. For the purified Ag_*N*_-DNA, binding of silver to oxygen atoms was present, suggesting that the interacting oxygens belong to the sugar moiety and/or phosphodiester bond. The crystal structures by Cerretani, *et al.*, found Ag atoms bound to the phosphate group, confirming this observation.^25,68,69^ In addition, Ag 3d core-level spectra were measured for various species containing both Ag(0) and Ag^+^. The 3d_5/2_ Ag peak shifts to higher binding energies (≃0.6 eV) as one goes from Ag(0) nanoparticles to Ag_*N*_-DNAs to Ag^+^–DNA complexes (Fig. 9), supporting an Ag_*N*_-DNA with a positive charge which is neither purely cationic nor fully reduced, in agreement with MS studies by others.^24,57,130^

**Fig. 10:**
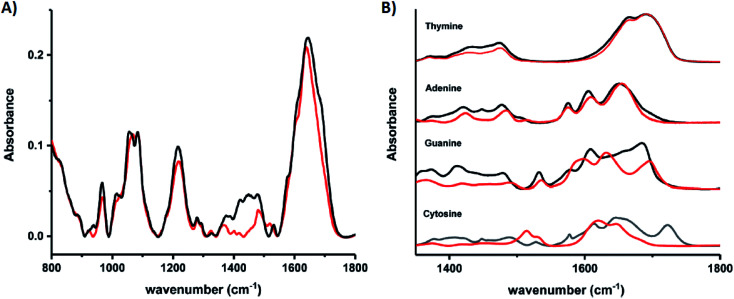
IR spectra of (A) bare DNA (black) and red-emitting Ag_*N*_-DNA (red) and (B) ss-DNA homopolymers of all four DNA nucleobases with (red) or without (black) Ag(i). Adapted from Schultz, *et al.*, (ref. 159) with permission from The Royal Society of Chemistry.

Schultz, *et al.*, recently studied an HPLC-purified Ag_*N*_-DNA^159^ emissive at 670 nm with a previously measured high quantum yield of 0.75.^24^ By combining analytical centrifugation with NMR and MS, it became apparent that despite HPLC isolation, the emissive product was a mixture of Ag_15_ and Ag_16_. Thus, even rigorous chromatographic separation may not always fully separate Ag_*N*_-DNAs into compositionally pure solutions when two or more species have very similar compositions/conformations. IR spectroscopy combined with MD simulations provided insights into the DNA binding sites of Ag^+^. The experimentally measured IR spectra of the Ag_*N*_-DNA and bare DNA only show marked shifts between 1350–1500 cm^−1^ after cluster formation (Fig. 10A). These shifts correspond to the nucleobases (Fig. 10B), not phosphate backbone, confirming that the Ag_*N*_ ligates primarily to DNA through Ag–nucleobase interactions.^54,55^

The Kohler and Petty groups very recently reported femtosecond time-resolved IR (TRIR) spectroscopic studies of two Ag_10_^6+^ clusters stabilized by very similar 18-base DNA strands, C_4_AC_4_TC_3_XT_3_, where X represents either guanosine or inosine (an artificial nucleoside lacking the exocyclic C2–NH_2_ of natural guanosine).^160^ These two DNA strands stabilize products with nearly identical spectra but dramatically differing quantum yields and fluorescence decay times, suggesting that the X nucleoside influences the excited state processes of the Ag_10_^6+^. Following excitation of the clusters by a 490 nm femtosecond laser pulse, the TRIR spectra are collected in the 1400–1720 cm^−1^ range, corresponding to spectral features from the nucleobases. While individual nucleobases are excited in the UV, TRIR spectra show that 490 nm excitation of the clusters results in bleaching of the vibrational modes of select nucleobases, most notably cytosine. Thymine appears unperturbed by cluster excitation, supporting the many past studies which show that silver has low affinity for this nucleobase at neutral pH and suggesting that this base does not coordinate with the cluster. Slight differences in the TRIR spectra of X = G and X = I Ag_*N*_-DNAs suggest that this method may enable precise probing of the electronic coupling of the Ag_*N*_ and surrounding nucleobases, a topic which remains poorly understood for Ag_*N*_-DNAs.^160^

### 3.4 Electron microscopy

Many reports of transmission electron microscopy (TEM) to characterize Ag_*N*_-DNAs report 2–20 nm particles, which have been attributed to the fluorescent clusters of interest.^161–170^ However, due to the much smaller sizes of Ag_*N*_-DNAs established by HPLC-MS and recent crystallographic studies (Section 3.5), it is highly likely that the particles observed in TEM are silver nanoparticles formed as byproducts during chemical synthesis.

### 3.5 Ag_*N*_-DNA cluster structures solved by X-ray crystallography

**Fig. 11:**
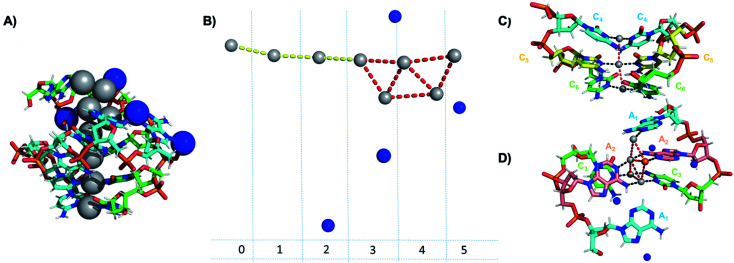
Asymmetric unit of the Ag_8_-DNA reported by Huard, *et al.*,^22^ with the 8-atom cluster (as defined in the authors' report) indicated by the gray spheres and additional silvers associated with crystal packing indicated by blue spheres. (B) Illustration of silver atoms only, for various crystal sections. (C and D) Structure of the Ag_8_-DNA in (C) Sections 0 to 2 and (D) Sections 3 to 5 as defined in (B). One silver atom (orange) is stabilized by an adenine from a neighboring DNA strand.^22^ Details on the structure can be found at the PDB database using accession code 6NIZ.

Crystallographic studies have recently begun to yield important insights into the structures of monolayer-protected silver clusters.^171–173^ Very recently, the first crystal structures have been reported for Ag_*N*_-DNAs.^22,25,68,69^ Huard, *et al.*, reported the first Ag_*N*_-DNA crystal structure for a cluster stabilized by two copies of a 6-base strand, 5′-AACCCC-3′.^22^ The asymmetric unit, shown in Fig. 11A, reveals a “Big Dipper” shape of 8 Ag atoms with planar geometry, identified as an Ag_8_-DNA. Four additional silvers not directly bound to the Ag_8_ promote crystal packing (blue spheres in Fig. 11). Ag–Ag distances in the pentameric core are ≃2.9 Å, comparable to Ag–Ag bond lengths in bulk silver.^174^ In this cluster core, adenines interact with Ag *via* N1 and N6, whereas cytosines are coordinated through N3 and N4 (Fig. 11). The exocyclic nitrogens (N4, N6) are hypothesized to be deprotonated. The zipper region is characterized by C–Ag^+^–C base pairs with parallel strand orientation and twisted base pairs, as observed elsewhere.^108,113^ Every Ag interacts with the N3 site of one cytosine on each strand (Fig. 11C), as established previously for Ag_*N*_-DNAs stabilized by C_12_ strands^52^ and for C–Ag^+^–C duplexes.^54,101^ All base-Ag interactions have distances of ≃2.1 Å. Unlike the Ag_*N*_-DNA studied by Schultz, *et al.*,^159^ significant interactions between adenines and Ag were found in the Ag_8_-DNA (Fig. 11D). It is notable that one Ag atom of the pentamer portion of the Ag_8_ is stabilized by a neighboring strand's adenine (Ag atom in orange in Fig. 11D), which may explain why the cluster could not be formed in solution without modifications to the DNA template strand.^22^

**Fig. 12:**
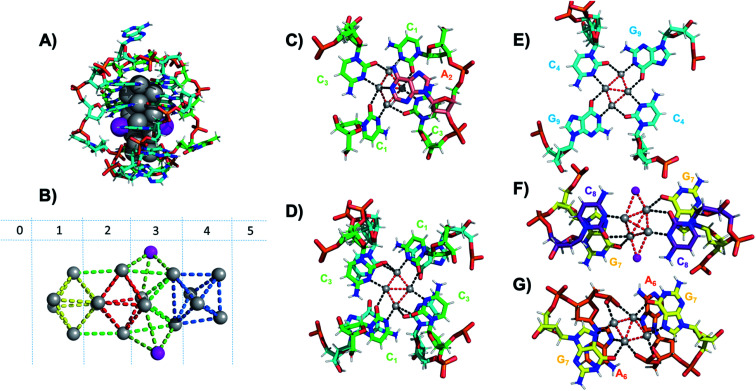
(A) Subunit structure of the Ag_16_-DNA (5′-CACCTAGCG-3′). Silvers with occupancy of 1 are gray, while lower occupancy silvers (∼0.3) are magenta. (B) Cluster structure with DNA removed, with sections numbered. (C) Sections 0 and 1, (D) Section 1 and a part of Section 2, and (E) Section 2 of the Ag_16_-DNA subunit. (F) Section 3, and (G) Sections 4 and 5 of the Ag_16_-DNA. Red dashed lines indicate Ag–Ag interactions, and black lines represent coordination bonds. Details on the structure of the NIR emissive Ag_16_-DNA can be found at the PDB database using accession code 6M2P. Adapted from Cerretani, *et al.*, (ref. 25) with permission from The Royal Society of Chemistry.

Six crystal structures have also been reported by Cerretani, *et al.*, for NIR Ag_16_-DNAs stabilized by DNA templates that differ by only one nucleobase.^25,68,69^ The first reported crystal structure is for an Ag_16_-DNA stabilized by two strands of a DNA decamer, 5′-CACCTAGCGA-3′, previously identified by Copp, *et al.*,^175^ with an unusually large Stokes shift.^176^ The second Ag_16_-DNA is stabilized by two copies of a 9-base sequence corresponding to removal of the A_10_ at the 3′-end of the decamer (Fig. 12A). Clusters formed on these two templates are nearly identical, and removal of the terminal A_10_ has no discernable impact on the wavelength of the absorbance peak but causes a slight redshift in fluorescence emission.^25^ Similarly, mutations of position 5 in the DNA sequence allow one to produce and crystalize a similar NIR emitter.^68^ The latter study showed also that certain nucleotide positions in the DNA sequence, while not relevant for binding to the Ag_*N*_, could be mutated in order to promote or alter crystal packing interactions. This concept could enable in the near future to re-engineer DNA sequences to promote crystallization and determine the structure of the emissive Ag_*N*_.^68^ It also demonstrates that, especially when the Ag_*N*_ is stabilized by multiple strands, the 3D organization of the nucleotides is more relevant than the sequential 5′ to 3′ order. Unlike the Ag_8_-DNA investigated by Huard, *et al.*, which did not perform NaBH_4_ reduction before the crystallization process, the Ag_16_-DNAs were synthesized in aqueous solution and then HPLC-purified prior to crystallization.

The clusters comprise 16 Ag atoms with occupancy of 1, along with additional silvers with lower occupancy (Fig. 12A and B). All bases, except thymine and one of the adenines in position 2, interact with Ag atoms, with the thymine ensuring strand flexibility and promoting crystal packing interactions (Fig. 12C–G). Most of the Ag–Ag distances are between 2.7 and 2.9 Å, similar to or shorter than their metallic radius. Nevertheless, the cluster charge cannot be elucidated by these distances alone and as mentioned previously, ample HR-MS data suggests that Ag_*N*_ clusters are generally highly cationic in nature.^23,24,57,130^ Similar to the crystal structure published by Huard, *et al.*,^22^ Ag atoms interact with cytosines *via* N3, and with adenines *via* N1. Interestingly, additional interacting sites were discovered, consistent with Schultz, *et al.*^159^ silvers coordinate O2 of cytosines, as well as N1, N7 and O6 of guanines, and N7 and the oxygens of the adenine phosphate group. Ag–N distances are 2.2–2.5 Å, mostly shorter than the Ag–O coordinate bond lengths 2.4 Å to 2.9 Å. The Ag–N bond lengths suggest that G_9_ is deprotonated at N1 (2.3–2.4 Å).^25,69^

Some Ag_*N*_-DNA crystal structures contain Ag^+^ which are not attached to the central cluster but do participate in non-WC base interactions and crystal packing.^22,25,68,69^ It is possible that such “accessory” Ag^+^ also exist in solution-phase Ag_N_-DNAs, as recently suggested by Gambucci, *et al.*^122^ If sufficiently tightly bound, these Ag^+^ would be counted by HR-MS as part of the Ag_*N*_-DNA but may not be part of the silver cluster itself and, thus, may not contribute significantly to the cluster's electronic properties. HR-MS results have not been reported for the Ag_8_ reported by Huard, *et al.*,^22^ nor the multiple Ag_16_ species reported by Cerretani, *et al.*,^25,68,69^ so it remains unknown whether all of the accessory Ag^+^ are present in solution. Studies which compare the MS-determined sizes of Ag_*N*_-DNAs with their crystallographic sizes are needed in order to probe the existence and role(s) of accessory Ag^+^ in Ag_*N*_-DNAs and, more generally, to what degree HR-MS measurements of purified Ag_*N*_-DNA species can discern the size of the emissive cluster. It will also be important to clearly state the assumptions made when assigning the cluster size *N* of an Ag_*N*_-DNA, particularly in light of the aforementioned evidence that observed optical properties are strongly correlated to the numbers of neutral silver atoms *N*_0_ determined by HR-MS and not necessarily the total silver atom number *N*_tot_.

### 3.6 Alternate possible cluster geometries and higher-order structures

We have primarily reviewed compositional and structural studies of Ag_*N*_-DNAs which are synthesized by chemical reduction and, notably, are stable under HPLC purification to enable accurate characterization. Smaller Ag_2_ and Ag_3_ clusters intercalated between base pairs of dsDNA can be synthesized by electrochemical means and exhibit ∼300 nm fluorescence emission,^177–180^ which supports smaller size and/or different cluster geometry than the HPLC-purified Ag_*N*_-DNAs discussed here. The versatility of macromolecular cluster ligands like DNA may permit multiple classes of metal clusters of the same metal species, even for Ag_*N*_-DNAs synthesized by chemical reduction. Thus, it is likely that other cluster sizes and geometries than the HPLC-purified ones discussed here may exist which may be unstable under the solvent and high-pressure conditions requisite for chromatographic separation.

### 3.7 Conformation of the DNA template strand

In addition to cluster structure, the secondary/tertiary structures of the cluster's DNA templates are of interest. An understanding of this structure is also critical for schemes which integrate with DNA nanotechnology.^181^ As before, we primarily review studies of purified samples or which employ techniques which may lead to isolated Ag_*N*_-DNA species, including microfluidic capillary electrophoresis,^49,182^ gel electrophoresis,^59,116^ and SEC.^38^

**Fig. 13:**
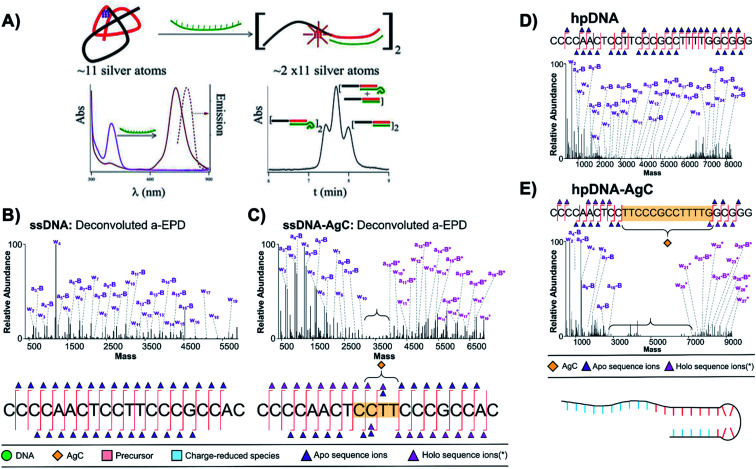
(A) Schematic of a sensor formed by a ∼11 Ag atom cluster with violet absorption, which converts into a NIR emissive cluster of twice the size upon hybridization with a target strand (green). Bottom right: Size exclusion chromatogram shows three separate peaks when a 10-thymine tail is appended to the target strand, indicating that the NIR Ag_*N*_-DNA forms by complexation of two DNA sensors.^38^ Adapted from Petty, *et al.*, (ref. 38) with permission from the American Chemical Society. Copyright 2013. (B–D) Deconvoluted a-EPD spectra and sequence coverage maps for a ssDNA template (B) without and (C) with an Ag_10_ and for a “hairpin” DNA template (D) without and (E) with an Ag_10_. Comparison of spectra with and without the Ag_10_ shows suppression of fragmentation for certain subregions of the DNA templates, which are correlated to regions where the DNA templates interact with their Ag_10_ clusters.^121^ Adapted from Blevins, *et al.*, (ref. 121) with permission from the American Chemical Society. Copyright 2019.

The Petty group combined SEC and other analytical methods to discern tertiary structures of their developed Ag_*N*_-DNA sensors, which signal binding of DNA analytes by transforming nonfluorescent Ag_11_ clusters on ssDNA templates into NIR-emissive clusters of twice the size.^38,183^ SEC separates complexes by molecular size and shape, with larger products eluting more quickly. A difference in retention time between two products indicates differences in molecular size. To count the number of DNA strands, *n*_s_, which scaffold the NIR cluster, 10-thymine tails were appended to one end of the target DNA analyte. SEC shows that a 1 : 1 mixture of DNA analytes with and without tails splits the chromatogram into three peaks. This splitting can be interpreted as complexation of two DNA probes to form the NIR Ag_*N*_-DNA (Fig. 13A). This was one of the first demonstrations of formation of Ag_*N*_-DNAs stabilized by template strand dimers,^38^ apart from HR-MS.^53^ For a modified sensor scheme, alignment of thymine tails was further used to probe alignment of the two DNA strands stabilizing the NIR Ag_*N*_-DNA.^183^ These clever experiments provide an alternate technique for inferring *n*_s_, which is especially useful for larger DNA complexes hosting an Ag_*N*_-DNA, which may not be stable even under gentle negative mode ESI-MS. Thymine tails were later used by Del Bonis-O'Donnell, *et al.*, to separate a set of Ag_*N*_-DNA-based probes for Hepatitis A, B, and C in a single microcapillary electrophoresis protocol.^184^

The Petty and Brodbelt groups recently determined and compared binding sites of two different Ag_10_ clusters to their DNA templates using activated electron photodetachment (a-EPD) MS.^121^ One Ag_10_ was stabilized by a 20-base strand which is single-stranded in the absence of silver (the subject of Fig. 8),^130^ and the other by a 28-base strand which forms a hairpin in the absence of silver (Ag_*N*_-DNAs were studied without subsequent purification).^157^ The DNA templates with and without Ag_*N*_ were analyzed by a-EPD, using 193 nm irradiation to induce DNA fragmentation, followed by MS. Fig. 13B–E compares mass spectra of the fragmented DNA host strands with and without Ag_*N*_, showing that certain fragments are suppressed in the presence of the Ag_*N*_. The suppression of fragmentation for certain regions of the DNA templates was associated with binding of the nucleobases to the Ag_*N*_ in these suppressed regions. For the ssDNA template, a remarkably short 4-base segment of CCTT was suppressed (Fig. 13C); in comparison to available crystal structures,^22,25,68,69^ it is reasonable that this segment represents only part of the silver-ligated nucleobases. For the hairpin DNA template, a much longer 13-base segment was suppressed, most of which is in the WC paired hairpin stem in the absence of silver (Fig. 13E). This provides credence to the notion that Ag^+^ can significantly reorganize DNA secondary structure. Future a-EPD studies could yield insights into other Ag_*N*_-DNAs.

## 4. Photophysical studies – probing excited luminescent and dark states of Ag_*N*_-DNAs

Compared to the current growing understanding of Ag_*N*_-DNA structure, the luminescence process of Ag_*N*_-DNAs remains less understood. Ag_*N*_-DNAs most certainly luminesce through an allowed fluorescence-like process, as supported by 1–4 ns fluorescence decay times and quantum yields >0.1 for most purified Ag_*N*_-DNAs.^24,123,176,185,186^ In contrast, phosphorescence-like emission of other metal clusters is characterized by much longer decay times and lower quantum yield values due to less allowed/forbidden transitions.^4,187^ However, the Ag_*N*_-DNA fluorescence process does differ from the simple Jablonski diagram of organic fluorophores.^188^ Ag_*N*_-DNAs lack the characteristic vibronic shoulders of organic molecular fluorophores,^21,147^ and their solvatochromic behavior is not well-described by Onsager-based methods used to model many organic fluorophores.^189^ Certain Ag_*N*_-DNAs retain surprisingly high quantum yields into the NIR,^190^ while quantum yields of organic dyes diminish rapidly in this region.^191^ Ag_*N*_-DNAs also have highly polarized excitation and emission due to well-defined transition dipole moments.^192^ Finally, the process of indirect fluorescence excitation *via* the DNA bases, which produces the same color of fluorescence as direct excitation in the visible or NIR excitation band of the Ag_*N*_-DNA (Fig. 1B),^21,26^ remains poorly understood. Here, we review spectroscopic studies of the photophysics of Ag_*N*_-DNAs, with a focus on purified Ag_*N*_-DNAs in more recent years. In order to ensure that measured photophysical properties are not affected by the presence of byproducts, such as Ag nanoparticles and nonfluorescent Ag_*N*_-DNAs, purification is essential to preparation and analysis of these fluorophores.

### 4.1 Ultrafast studies of the Franck-Condon state

**Fig. 14:**
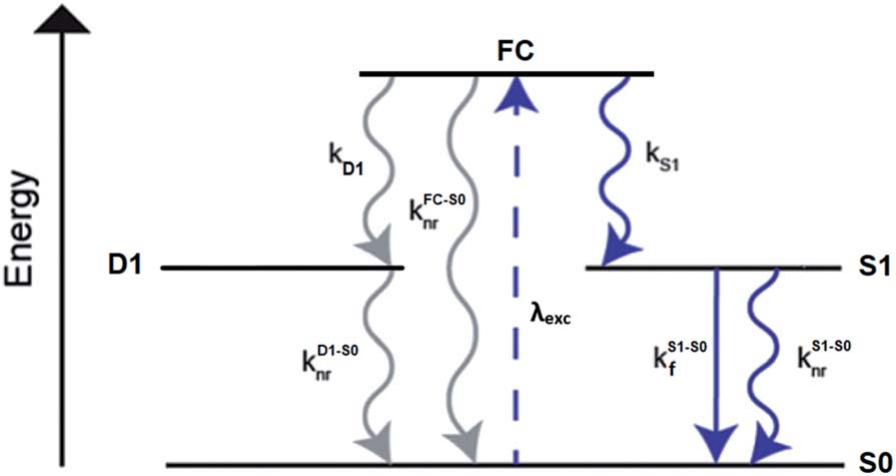
General phenomenological model for Ag_*N*_-DNAs. S0 and S1 represent the ground and emissive states, respectively, FC indicates the initially populated Franck-Condon state, and D1 is the dark state. The dashed blue line stands for the absorption process, wavy lines represent non-radiative pathways, and the straight line defines the emissive decay. Adapted from Cerretani, *et al.*, (ref. 185) with permission from the Royal Society of Chemistry.

**Fig. 15:**
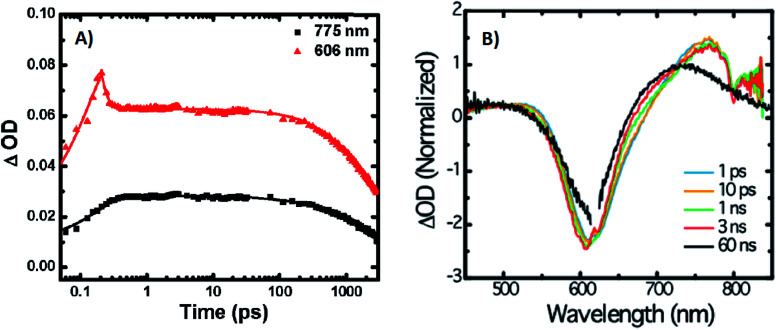
(A) Femtosecond transient absorption kinetic traces for 680 nm emissive (“Ag680”) Ag_*N*_-DNAs. The wavelengths shown for this emitter reflect the transient absorption (black) and the ground-state depletion (red). The depletion appears at negative ΔOD, but is plotted in its absolute value. It has been corrected for the spectral overlap by subtracting the contribution from the transient absorption, which is based on the kinetics at 775 nm calibrated to the expected value based on the peak curve fittings. The data was collected by exciting with a 100 fs Ti-sapphire laser at 1 kHz, then probing with a white light continuum generated from the same laser. The excitation wavelength was tuned to the peak of the ground state absorption. (B) Normalized femtosecond and nanosecond transient absorption spectra for Ag680. The sample was excited by 100 fs pulsed excitation, except for the long delay time curve, which was generated from excitation by a 7 ns pulsed laser. The dip in the spectrum around 800 nm is an instrumental artifact.^193^ Adapted from Patel, *et al.*, (ref. 193) with permission from the American Chemical Society. Copyright 2009.

A limited number of experimental studies have probed the ultrafast dynamics that occur upon excitation of Ag_*N*_-DNAs to the initial excited state (Franck-Condon state).^26,27,193,194^ Patel, *et al.*, proposed the first phenomenological model describing the excitation process (Fig. 14), based on ultrafast transient absorption experiments performed on three unpurified red and NIR Ag_*N*_-DNAs (Fig. 15).^193^ It was observed that a fraction of the population in the Franck-Condon state returned to the ground state with a time constant in the hundreds of fs, as seen by the ground-state recovery (Fig. 15A). Additionally, a rise component of similar time scale was attributed to formation of the emissive state. This emissive state then decays back to the ground state on a nanosecond timescale, as witnessed by similar time-scales of the ground-state recovery and the typical ns fluorescence decay times measured by time-correlated single photon counting (TCSPC).^35,176,185^ In addition, nanosecond transient absorption spectroscopy (Fig. 15B), together with single molecule blinking experiments,^8^ also showed the presence of a dark state with a μs-scale decay time. To date, no significant emission from this dark state has been observed, indicating that it decays mainly nonradiatively back to the ground state. Whether the dark state originates directly from the Franck-Condon state or is formed from the emissive state can be determined by the rise time of the dark state formation itself. Nanosecond transient absorption experiments were performed to determine the rise time of the dark state. These experiments concluded that dark state formation was limited by the instrument response function (IRF) of 7 ns, prohibiting discernment of the state from which the dark state is formed. Recent single molecule results by Krause, *et al.*, yielding secondary fluorescence (emission generated from OADF) over primary fluorescence intensity ratios higher than 1, suggest that the dark state is formed from the initial Frank–Condon state.^195^

Recently, Thyrhaug, *et al.*, performed 2D electronic spectroscopy experiments^27^ on a previously sized NIR-emitting Ag_20_-DNA.^53,196^ Excitation into the Franck-Condon state led to ultrafast evolution of the Franck-Condon state into the emissive state, which then decayed on a nanosecond time-scale observed from TCSPC measurements. The transfer from the initially populated state to emissive state occurred in about 140 fs, in line with the order of magnitude reported by Patel, *et al.*^193^ Additionally, the Ag_*N*_-related absorption feature appeared to consist of two closely lying transitions, and a coherent excitation of both states occurred due to the short pulse width of the laser. Interestingly, for this particular Ag_*N*_-DNA, coherence was transferred to the emissive state and can be seen by oscillatory quantum beating features that dephased with a time constant of ∼800 fs. Thus, after a few ps, all ultrafast processes were complete, and the only remaining process is the ∼ns fluorescence. The dominant quantum beating mode frequency of 105 cm^−1^ is similar to Ag–Ag vibrational modes.^197^

### 4.2 Dark states

The presence of a μs-lived dark state in Ag_*N*_-DNAs was first reported by Vosch *et al.*^8^ Ag_*N*_-DNAs were immobilized in a polyvinyl alcohol (PVA) film and fluorescence intensity was recorded as a function of time. Autocorrelations of the fluorescence intensity trajectories revealed μs blinking. A similar μs correlation time was observed by fluorescence correlation spectroscopy (FCS) in solution. FCS experiments are not only useful for determination of the decay time of the dark state and the quantum yield of dark state formation^198^ but also for estimation of the molar extinction coefficient by determining the number of emitters in a certain volume identified from a reference measurement.^8,199^ While nontrivial to determine or suggest the exact nature of the dark state, dark states have been reported in other studies^60,196,200^ and may be common for most Ag_*N*_-DNAs. The quantum yields of dark state formation have been estimated to range from a few up to 25 percent.^8,60,196,201^ When removal of molecular oxygen from the environment results in a lengthening of the dark state decay time, this is often a good indicator that the dark state is a triplet state.^202^ For Ag_*N*_-DNAs, the DNA scaffold around the silver cluster might act as a physical barrier for this type of Dexter-type triplet state quenching, resulting in minimal or no effect of removal of oxygen on the dark state decay time.^8^

**Fig. 16:**
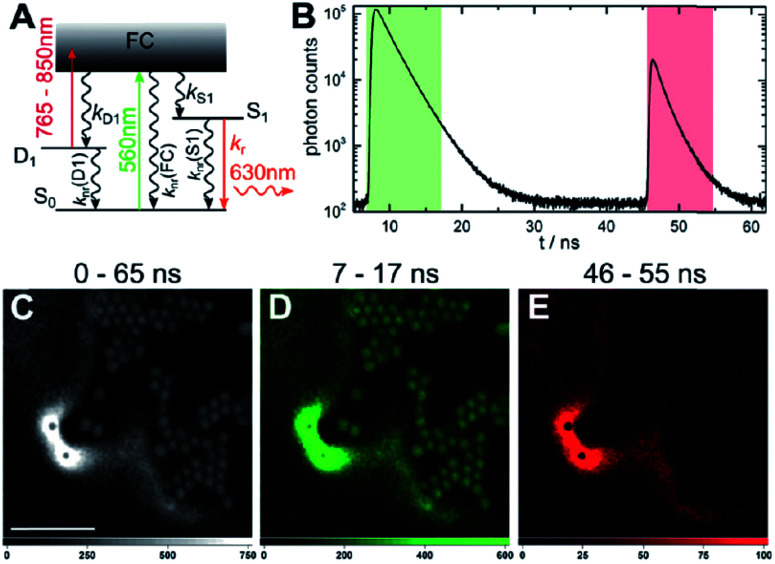
OADF microscopy. (A) Energy diagram for OADF of a red emissive Ag_*N*_-DNA. Vertical colored arrows indicate absorption of a photon from primary (560 nm) and secondary (765–850 nm) excitation lasers and fluorescence emission at 630 nm, respectively. (B) Primary fluorescence decay curve (first decay after excitation with 560 nm at 7 ns) and OADF decay (second decay after illumination with 765–850 nm at 46 ns) for red Ag_*N*_-DNA embedded in PVA. (C–E) Fluorescence images of a heterogeneous sample of fluorescently-labeled polystyrene microspheres, which are auto-fluorescent to simulate undesired background, and red-emitting Ag_*N*_-DNAs within PVA film (the signal of interest). Images were constructed using (C) all detected photons (0–65 ns), (D) primary fluorescence (7–17 ns) and (E) OADF signal (46–55 ns). Scale bar corresponds to 10 μm. The time gates used to construct images (D) and (E) are shown in (B) with the same colors. Images acquired with 3.7 kW cm^−2^ primary excitation power.^30^ Adapted from Krause, *et al.*, (ref. 30) with permission from the Royal Society of Chemistry.

Richards, *et al.*, demonstrated that the dark state formed by a primary excitation laser can be optically excited with a secondary NIR laser, resulting in depletion of this long-lived state and an overall increase in fluorescence intensity.^31^ It was recently proven that optical excitation of the dark state can transition the Ag_*N*_-DNA to the emissive state, resulting in optically activated delayed fluorescence (OADF).^28–30,195^ This process is similar to typical reverse-intersystem crossing processes observed in organic dyes.^203,204^ OADF combined with time-gating provides background-free signal because the delayed fluorescence is on the Anti-Stokes side (lower wavelength side) of the secondary excitation laser, allowing any Stokes-shifted auto-fluorescence from the secondary laser to be suppressed with a short-pass filter in the detection path. Fig. 16 shows an example by Krause, *et al.*, that demonstrates the OADF imaging concept.^30^ Additionally, Krause, *et al.*, showed that the use of the secondary NIR laser only (blocking the primary excitation laser) yielded similar fluorescence which was linearly dependent on the excitation intensity. This process is termed upconversion fluorescence (UCF),^29,30^ in analogy to the well-established upconversion processes in lanthanide based emitters.^205^

### 4.3 Emissive state

**Fig. 17:**
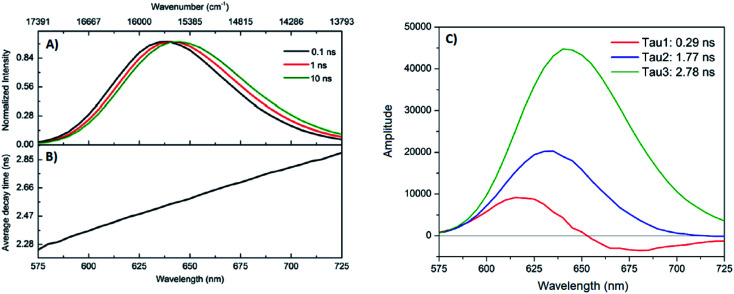
(A) TRES, (B) average decay time as a function of emission wavelength, and (C) DAS of red emissive Ag_*N*_-DNAs at 25 °C, excited at 561 nm. The gray line in (C) indicates the zero line. In order to construct TRES and DAS, the intensity decays were acquired from 575 nm to 725 nm, in steps of 5 nm. The three decay time values were globally linked in the fit.^185^ Adapted from Cerretani, *et al.*, (ref. 185) with permission from the Royal Society of Chemistry.

While one would expect a single emissive species to exhibit mono-exponential fluorescence decay, several HPLC-purified Ag_*N*_-DNAs with long DNA template strands (19–30 bases) exhibit multi-exponential fluorescence decay.^35,185,186^ Because solutions are purified prior to characterization and no shift is present in the steady-state emission as a function of excitation wavelength, a heterogeneous mixture of Ag_*N*_-DNA species can be excluded as the cause of this multi-exponential decay. The multi-exponential decay behavior can instead be explained by relaxation of the emissive state on a time-scale similar to the fluorescence decay. This effect, termed “slow” spectral relaxation, can be confirmed by time-resolved emission spectra (TRES) which show a gradual red-shift of the emission maximum on the nanosecond time scale (Fig. 17A). We note that the “slow” spectral relaxation is a minor part of the overall Stokes shift, with the majority of relaxation occurring on a time-scale below the IRF. A consequence of “slow” spectral relaxation is that the average decay time increases as a function of emission wavelength (Fig. 17B). Furthermore, the decay associated spectra (DAS) usually lead to spectra where the fastest decay time component tends to have positive amplitudes at shorter wavelengths and negative amplitudes (rise) at longer wavelengths (Fig. 17C).^185^ Only two processes can cause this effect: energy transfer or “slow” spectral relaxation.^188^ Energy transfer can be excluded since, as stated above, there is no evidence for multiple independent emitters in the HPLC-purified Ag_*N*_-DNA solutions.

Unlike small solvent molecules which rearrange on picosecond timescales, the DNA template and its structurally bound water molecules require much longer, up to a few nanoseconds, to adapt to the new charge distribution of the Ag_*N*_-DNA in the emissive state. A similar effect was observed when a coumarin dye was embedded in an abasic site of dsDNA.^206^ Emission spectral shifts could be observed from the femtosecond time scale up to tens of nanoseconds. Other parameters, *e.g.* changes to solvent viscosity or temperature, also affect the “slow” spectral relaxation.^176,185^

If spectral relaxation occurs entirely within the time-scale of the IRF, the observed decay time will be mono-exponential. This is the case for Ag_*N*_-DNAs stabilized by short, 9–10 base DNA strands, whose “slow” spectral relaxation is negligible at room temperature and in low viscosity solvents,^25,176,190^ most likely because multiple short strands are more flexible and rearrange faster than one long oligomer. Spectral relaxation could be a useful tool to establish the rigidity of the DNA scaffold and its effect on the excited state of Ag_*N*_-DNAs.^35,51,185,186^

### 4.4 Excitation and emission transition dipole moments

Another interesting spectroscopic feature of Ag_*N*_-DNAs is their parallel excitation and emission transition dipole moments. Hooley, *et al.*, employed defocused widefield microscopy to investigate the transition dipole moments of a C_24_-templated Ag_*N*_-DNA immobilized in PVA.^192^ By defocusing a common widefield image, the emission of a single emitter displays a bilobed shape that depends on the orientation of its emission transition dipole. In order to determine both excitation and emission transition dipoles simultaneously, defocused widefield microscopy was combined with rotating the polarized excitation light. Then, the intensity of each emitter is directly correlated to the excitation efficiency of the Ag_*N*_-DNA. Maximum emission intensity was observed when excitation light was aligned with the emission transition dipole, indicating that the excitation and emission transition dipole moments lie along a similar direction.

**Fig. 18:**
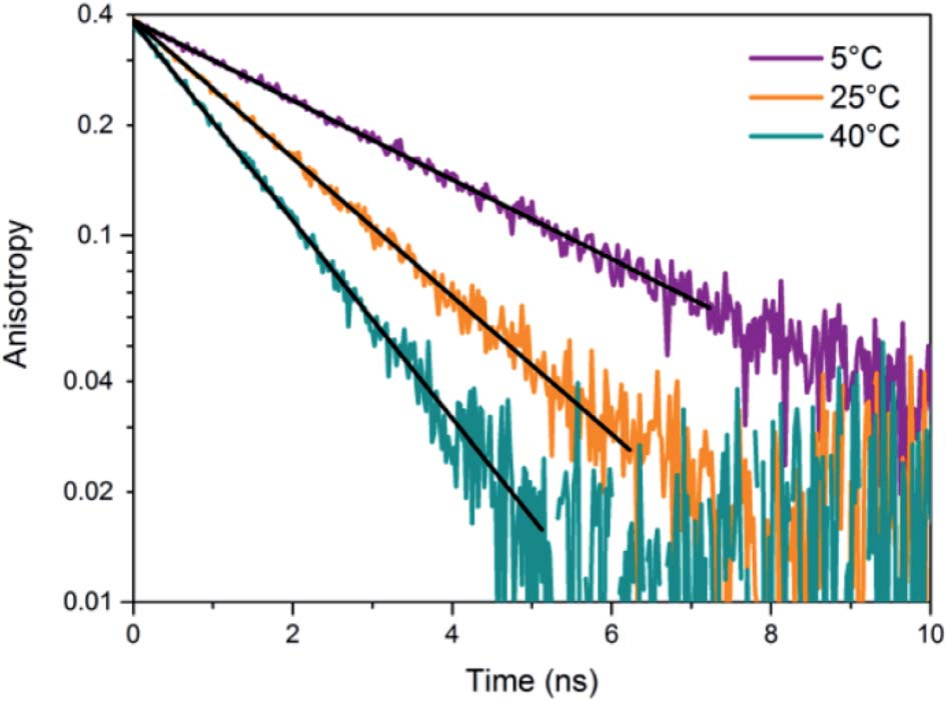
Anisotropy decays of a NIR Ag_*N*_-DNA in 10 mM NH_4_OAc aqueous solution at 5 °C, 25 °C, and 40 °C. Data was fitted assuming a single rotational correlation time.^176^ Adapted from Bogh, *et al.*, (ref. 176) with permission from the Institute of Physics.

The Vosch group has also observed further evidence of the alignment of excitation and emission transition dipole moments by time-resolved anisotropy measurements.^122,176,190^ Three different Ag_*N*_-DNAs, two NIR emitting and one red, displayed limiting anisotropy values close to 0.4, which indicates that the excitation and emission transition dipole moments are parallel (one example in Fig. 18).

### 4.5 Coherent two-photon excitation

Patel, *et al.*, first reported two-photon excitation (800 to 1000 nm range) of Ag_*N*_-DNAs in 2008, in a study of four non-purified Ag_*N*_-DNAs with emission maxima at 620 nm, 660 nm, 680 nm and 710 nm.^207^ For the 660 nm, 680 nm and 710 nm emitters, the two-photon emission exhibited quadratic dependency on excitation intensity, as expected, and one-photon and two-photon fluorescence decay times were similar, indicating that emission occurred from the same emissive state. The one *versus* two-photon excitation spectra of the 620 nm emitter indicated that cross-section maxima occurred at different wavelengths. The reported two-photon cross-sections ranged from 33 900 to 50 000 GM, roughly two orders of magnitude higher than typical organic fluorophores (*e.g.* 210 GM at 840 nm for Rhodamine B).^208^ Yau, *et al.*, reported a two-photon cross-section of ∼3000 GM at 800 nm and quadratic dependence of emission on excitation intensity for an unpurified 650 nm emitter.^194^ This 650 nm emitter was made by first creating an Ag_*N*_-DNA using ssDNA, followed by addition of an excess of a complementary strand with a guanine-rich section. Because few studies have probed two-photon excitation of Ag_*N*_-DNAs, future investigations on purified Ag_*N*_-DNA could shed light on the origin of the very high two-photon cross-sections.

## 5. Informed design – decoding the sequence-color connection for Ag_*N*_-DNAs

The fascinating sequence dependence of Ag_*N*_-DNAs results from the nucleobase-specific interactions of DNA with silver (Section 2). The ability of DNA sequence to select for the sizes and optical properties of metal nanoclusters has attracted great interest due to the promise of highly customized fluorophores.^199,209^ To date, it is likely that thousands of different DNA template strands have been reported, corresponding to Ag_*N*_-DNAs with wide-ranging fluorescence colors, Stokes shifts, quantum yields, chemical yields, photostabilities, and chemical stabilities.^70^ Yet the connection between DNA sequence and Ag_*N*_-DNA properties has remained obscure. Most studies select Ag_*N*_-DNAs by experimentally testing small numbers of DNA template strands rich in C or G.^32,46,128^ One large-scale study by the Dickson group used DNA microarrays to identify fluorescent Ag_*N*_-DNAs, but only a few of the DNA template sequences were reported.^199^ To fully realize Ag_*N*_-DNAs as programmable materials, it is crucial to “decode” the connection between DNA sequence and Ag_*N*_-DNA properties.

Rational design of Ag_*N*_-DNAs is especially challenging due to an astronomical number of possible DNA template sequences and a complex connection between Ag_*N*_-DNA color and DNA sequence. Ag_*N*_-DNA templates are typically 10–30 base oligomers. Because a sequence of the four natural nucleobases can have 4^*L*^ distinct *L*-base sequences, Ag_*N*_-DNA templates must be chosen from 4^30^ (∼10^18^) possible sequences. While in some cases subtle sequence changes can dramatically shift fluorescence,^65,169^ in other cases different DNA sequences can stabilize Ag_*N*_-DNAs with the same emission wavelength.^57^ To make matters more complex, some DNA sequences can stabilize different types of fluorescent Ag_*N*_ clusters,^57^ with yields of each cluster species possibly depending on synthesis method and/or Ag:DNA stoichiometry. First-principles computational methods have not yet matured sufficiently to model the structures of realistic Ag_*N*_-DNAs, let alone their accurate electronic properties. Small-scale studies of DNA sequences with constrained patterns^119,169,196,210–212^ have been useful for developing a few Ag_*N*_-DNAs with well-controlled properties but are limited in their applicability to the majority of reported Ag_*N*_-DNAs. Here, we review large-scale experimental studies of the Ag_*N*_-DNA sequence-color connection for 10^3^ DNA strands, in which machine learning enables predictive design and provides new physical insights.

### 5.1 Large-scale studies of sequence dependence

The combinatorial nature of DNA makes data science well-suited to study how DNA sequence selects Ag_*N*_-DNA properties. Copp, *et al.*, have pioneered high throughput experiments together with supervised machine learning (ML) to understand how DNA sequence selects for Ag_*N*_-DNA fluorescence emission and to predict new templates for optimized Ag_*N*_-DNAs. The methods described here have uncovered Ag_*N*_-DNAs which are the subjects of later detailed studies.^23,69,176,190^ For readers seeking to learn more about ML, we recommend a tutorial review by Domingos^213^ and a review of ML for soft matter by Ferguson.^214^

To train a ML algorithm to output Ag_*N*_-DNA fluorescence properties (or whether *any* fluorescent product can be stabilized) given an input DNA template sequence, one must first amass a data library connecting DNA sequence to Ag_*N*_-DNA fluorescence spectra for hundreds to thousands of sequences. This data cannot be mined from the literature because (i) synthesis and characterization methods vary widely, prohibiting isolation of the effects of DNA sequence from other experimental parameters, and (ii) while ∼75% of DNA sequences are unsuitable for templating fluorescent Ag_*N*_-DNAs, these “negative” DNA sequences are rarely reported.^57^ The absence of negative sequences from the literature is problematic because to effectively learn what makes a suitable DNA template for brightly fluorescent Ag_*N*_-DNAs also requires knowledge of what does not make a suitable template.

**Fig. 19:**
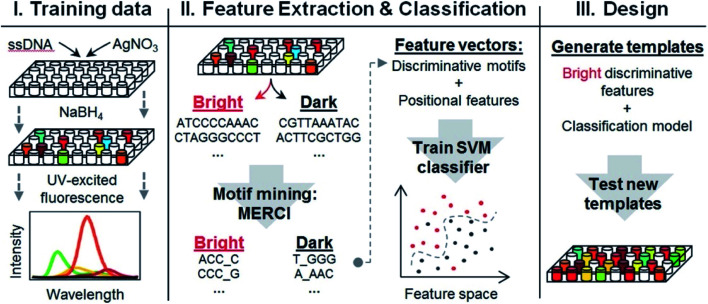
Schematic of the workflow for supervised learning applied to prediction of DNA template sequences for brightly fluorescent Ag_*N*_-DNAs.^215^ Adapted from Copp, *et al.*, (ref. 215) with permission from John Wiley and Sons. Copyright 2014.

To enable ML for Ag_*N*_-DNAs, Copp, *et al.*, developed high-throughput Ag_*N*_-DNA synthesis and characterization in well plate format using robotic liquid handling followed by rapid fluorimetry,^57^*via* universal UV excitation of all Ag_*N*_-DNA products through the nucleobases (Fig. 19 part I).^21^ Because fluorimetry is performed one day, one week, and four weeks after synthesis, this data set allows ML studies to focus only on time-stable Ag_*N*_-DNAs. Experiments are normalized using a well-studied Ag_*N*_-DNA control,^24,35,122,185^ for direct comparison of fluorescence wavelength and intensity among all data in the library. To date, we have reported on >3000 distinct DNA template sequences, most 10 bases long, for Ag_*N*_-DNA synthesis in 10 mM NH_4_OAc aqueous solution of neutral pH.^23,111,175,215^

Effective ML requires appropriate choice of “feature vectors,” which are the parameterizations of training data provided as inputs to the ML classifier(s). For Ag_*N*_-DNAs, feature vectors should represent the salient properties of a DNA sequence which determine how sequence is mapped onto Ag_*N*_-DNA fluorescence. Because these properties are not well-known (otherwise ML would be unnecessary), this feature engineering process is a critical step in the ML workflow^213^ and has led to new physical insights into Ag_*N*_-DNAs. Early work used training data for 684 randomly generated 10-base DNA sequences to learn to predict Ag_*N*_-DNA fluorescence brightness given an input template strand sequence.^215^ Using integrated fluorescence intensity, *I*_int_, as a metric of brightness, sequences with the top 30% of *I*_int_ values were defined as bright and the bottom 30% of *I*_int_ values defined as “dark.” Then, a ML algorithm called a support vector machine (SVM) was trained to distinguish bright and dark sequences (Fig. 19 part II). It was found that the SVM most accurately predicted a sequence's class if feature vectors were engineered to quantify the occurrence of certain DNA subsequences called “motifs” which were identified by bioinformatics approaches to be correlated with one class but not the other.^216^ The resulting trained SVM's classification accuracy was 86%, as determined by cross-validation (a process which trains on most of a training data set and reserves a small ∼10% portion as a “test set” to assess SVM performance on data which the ML classifier has not yet encountered). New DNA templates for bright Ag_*N*_-DNAs were designed using the bright-correlated motifs as building blocks and then screened by the trained SVM to choose those predicted as most likely to be bright. 78% of designed DNA templates stabilized bright Ag_*N*_-DNAs, as compared to 30% of the initial random sequences.^215^ This early work pointed to the role of certain DNA base motifs in stabilizing Ag_*N*_-DNAs, which agreed with later findings that not all DNA bases coordinate the Ag_*N*_.^22,25,69^

**Fig. 20:**
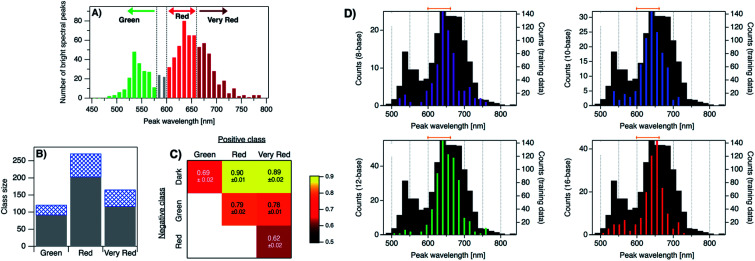
(A) Distribution of peak fluorescence emission wavelength for Ag_*N*_-DNAs stabilized by ∼2000 different 10-base DNA templates, with arrows and colors indicating the color classes defined in the text. (B) Numbers of DNA sequences in color classes from (A), corresponding to samples with bright spectral peaks in only one class (gray). Other sequences exhibited secondary bright peaks in a different color class (checkered blue) but were omitted from the training data in order to best learn the features of DNA sequences suitable for only one size of fluorescent Ag_*N*_-DNA. (C) Cross-validation scores for trained one-*versus*-one SVMs. The color bar indicates score value, which is also indicated by text on each pixel.^175^ (A–C) Adapted from Copp, *et al.*, (ref. 175) with permission from the American Chemical Society. Copyright 2018. (D) Distributions of observed fluorescence peaks for 10-base Ag_*N*_-DNA training data (black) and Ag_*N*_-DNAs designed by ML (colored bars) for Red 600–660 nm fluorescence (target color band indicated by orange brackets). Designed DNA template strand lengths vary: 8 bases (purple), 10 bases (blue), 12 bases (green), and 16 bases (red).^111^ Adapted from Copp, *et al.*, (ref. 111) with permission from the American Chemical Society. Copyright 2020.

While predicting Ag_*N*_-DNA fluorescence intensity increases the likelihood of selecting fluorescent Ag_*N*_-DNAs by three-fold, this simple method also prefers red-fluorescent Ag_*N*_-DNAs over green Ag_*N*_-DNAs.^215^ It is ideal to instead predict both brightness and color from an input DNA sequence. To achieve this, Copp, *et al.*, used physically motivated Ag_*N*_-DNA classification based on the known correlation between Ag_*N*_-DNA color and cluster size. The multi-modal distribution of Ag_*N*_-DNA fluorescence colors in the visible spectrum was shown to arise due to the magic numbers of these clusters: Ag_*N*_-DNAs in the 500–570 nm abundance have *N*_0_ = 4 neutral Ag atoms, while Ag_*N*_-DNAs in the 600–670 nm abundance have *N*_0_ = 6 (Fig. 20A).^57^ Because “Green” and “Red” Ag_*N*_-DNAs have distinct cluster sizes, there is likely a fundamental difference between template sequences for these two cluster sizes. To learn to distinguish between DNA sequences based on cluster structural differences, a training data set of ∼2000 10-base DNA sequences was separated into four color classes: the three shown in Fig. 20A (“Very Red” is defined as the high wavelength histogram shoulder, which may signal a different cluster structure) and a “Dark” class similar to the one previously defined.^215^ Because the numbers of sequences in these classes are unequal, with far more Dark sequences than Green sequences (Fig. 20B), it is critical to apply subsampling to balance classes prior to ML, ensuring training on equal numbers of sequences from each class.^217,218^ Feature vectors were then constructed using DNA motif mining to identify color-correlated motifs, followed by feature selection^219^ to reduce the list of selected motifs to those most important for classification; this critical step reduces problems which can arise from overfitting. We note that both data balancing and feature selection should generally be applied when using ML for real-world materials systems.

Because SVMs are inherently binary classifiers, a “one-*versus*-one” approach was used to distinguish the four color classes. Six different SVMs were trained to discriminate between the six possible pairs of classes (cross-validation scores, which represent the accuracy of classification, in Fig. 20C). To experimentally test the performance of the trained classifiers, new DNA template sequences were designed for the two least abundant classes, Green and Very Red. First, color-correlated DNA motifs for the desired class were selected from a probability distribution weighted by intensity and placed into an initially empty DNA sequence. Second, designed candidate DNA templates were screened by the trained SVMs to estimate the probability of falling within the desired color class. Finally, templates corresponding to the top 180 probabilities were selected for experimental testing. With this method, the likelihood of selecting a Very Red Ag_*N*_-DNA increased by nearly 330%, and the likelihood of selecting a Green Ag_*N*_-DNA was increased by >80%.^175^ This method was later modified to enable design of Ag_*N*_-DNA templates of any strand length, and it was found that training data of only 10-base sequences still enabled effective prediction of Ag_*N*_-DNA color for other lengths of DNA templates, up to the maximum 16-base length tested (Fig. 20D).^111^ This suggests that there exist certain DNA motifs which are selective of cluster type and thus color for a range of DNA template lengths, making ML design approaches for Ag_*N*_-DNAs much more promising. We note that thus far, all Ag_*N*_-DNAs stabilized by DNA templates of <19 bases have been found to be “strand dimers” which contain two template strands per cluster;^23,24,53,57^ it is possible that longer DNA templates, which have not been designed by ML, may have some different DNA sequence rules for Ag_*N*_-DNA color selection.

**Fig. 21:**
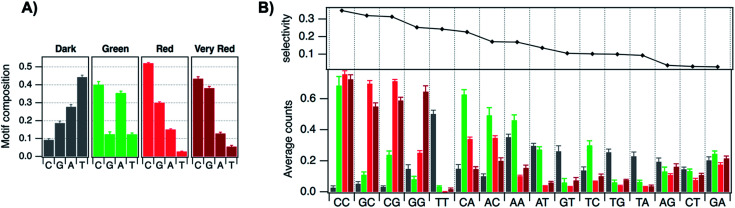
Average numbers of (A) single bases and (B) two-base patterns in motifs identified by feature selection to be correlated to Ag_*N*_-DNA color (bar color indicates sequence class: gray = Dark, green = Green, red = Red, dark red = Very Red). In (B), two-base patterns are ordered along the horizonal axis by selectivity, defined to be the standard deviation of the heights of the four bars for each base pattern.^175^ Adapted from Copp, *et al.*, (ref. 175) with permission from the American Chemical Society. Copyright 2018.

In addition to improving design efficiency, ML provides key insights into how DNA sequence selects silver cluster size, and thus fluorescence wavelength. Fig. 21B shows average base composition of the motifs identified by feature selection to be most predictive of Dark, Green, Red, and Very Red sequences.^175^ To summarize, thymines are strongly correlated with no fluorescence. Adenines show preference for smaller and Green Ag_*N*_-DNAs, while guanines, particularly consecutive guanines, are correlated with long wavelength fluorescence (associated by MS with clusters containing more Ag atoms). Cytosines are strongly selective for fluorescence brightness but less selective of color than A or G. To better understand these correlations, we compare to HR-MS studies of DNA-Ag^+^ complexes (Section 2), which are the precursors of Ag_*N*_-DNAs prior to reduction by NaBH_4_. Fig. 3A shows the distribution of Ag^+^ attached to single DNA homobase strands or pairs of strands, and Fig. 5A shows the same distribution for Ag^+^-mediated dimers of C or G strands with central base mutations.^54,55^ Because homo-thymine strands only weakly associate with Ag^+^, thymine-rich DNA sequences may be unsuitable (at neutral pH) to host fluorescent Ag_*N*_ due to (i) too few Ag atoms recruited prior to reduction, resulting in insufficient silvers to form a cluster and/or (ii) little to no coordination with the cluster. The greater occurrence of T's in green Ag_*N*_-DNA templates further supports this notion, since these clusters are smaller in size and may require fewer nucleobase coordination sites. Adenine homobase strands bind to a few Ag^+^, which may support formation of smaller *N*_0_ = 4 clusters with green emission. In comparison to A and T, C- and G-rich homobase strands can form Ag^+^-mediated duplexes with ∼1 Ag^+^ per base pair, providing more Ag atoms during cluster growth and supporting nucleobase–silver binding in the Ag_*N*_-DNA. Interestingly, duplexes of G homobase strands with a single central A, C, or T base mutation can harbor ∼60% more Ag^+^ than G homobase polymers with no mutation. This significant increase in Ag^+^ attachment as compared to C-rich strands, and the structural differences in the DNA secondary/tertiary structures supported by IMS-MS of these strands,^108^ could explain why consecutive G's are strongly associated with Very Red Ag_*N*_-DNAs.^175^

## 6. Supra-cluster assembly – towards applications in photonics and sensing

Structural DNA nanotechnology harnesses DNA as a programmable building block for self-assembled nanostructures.^220^ It is promising to combine sequence-controlled Ag_*N*_-DNAs with DNA nanotechnology for realization of precise metal cluster arrays, which could be envisioned as functional sensors and photonic devices. These achievements will require robust strategies to effectively embed Ag_*N*_-DNAs into larger WC-paired architectures. Here, we review efforts to harness DNA self-assembly for multi-Ag_*N*_-DNA organization (many groups have incorporated single Ag_*N*_-DNAs within WC paired DNA structures to build biomolecular sensors,^13,14,33,37,73,183^ which were recently reviewed elsewhere^74^).

**Fig. 22:**
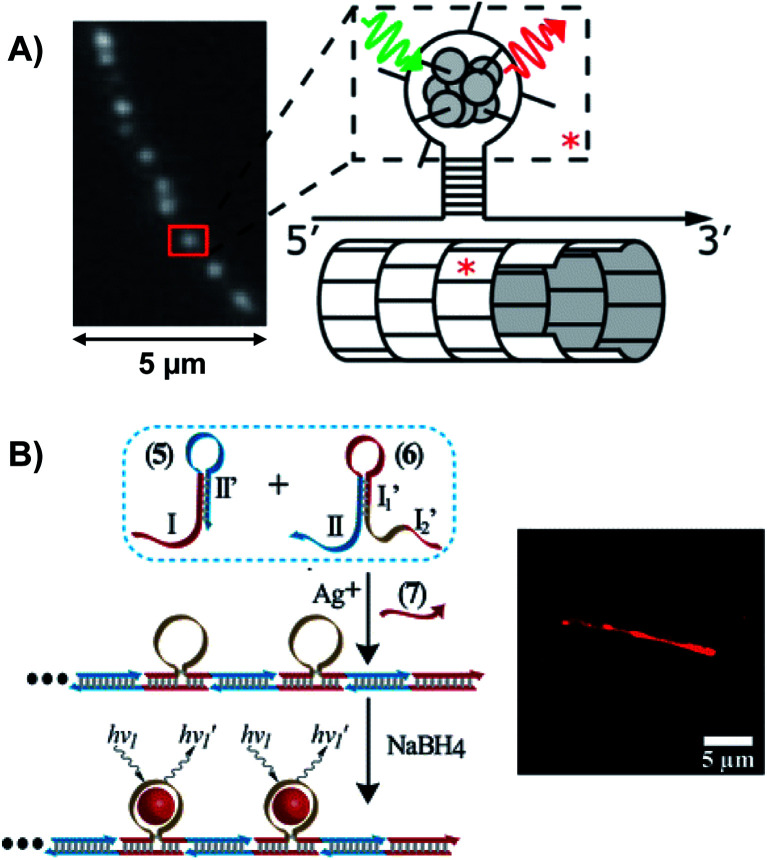
(A) Schematic and fluorescence micrograph of an Ag_*N*_-DNA-labeled DNA nanotube, with clusters templated by hairpin protrusions on select DNA tiles (red asterisk).^100^ Adapted from O'Neill, *et al.*, (ref. 100), with permission from the American Chemical Society. Copyright 2012. (B) Schematic of hybridization chain reaction (HCR) forming wire scaffolds for Ag_*N*_-DNA synthesis, and fluorescence micrograph of synthesized Ag_*N*_-DNA wire.^118^ Adapted from Orbach, *et al.*, (ref. 118) with permission from the American Chemical Society. Copyright 2013.

O'Neill, *et al.*, first reported decoration of a DNA nanostructure with multiple Ag_*N*_-DNAs.^100^ A mixture of green and red clusters were synthesized onto ssDNA hairpin protrusions programmed into DNA nanotubes (Fig. 22A). Without hairpins, nanotubes did not foster cluster growth, consistent with early findings that dsDNA is an unsuitable Ag_*N*_-DNA template.^11^ The authors noted that Tris buffers typical of DNA self-assembly schemes were unsuitable for chemical synthesis of fluorescent Ag_*N*_-DNAs; this incompatibility is commonly faced in supra-cluster assembly of Ag_*N*_-DNAs. Orbach, *et al.*, demonstrated Ag_*N*_-DNA synthesis on μm-scale DNA wires with hairpin protrusions.^118^ Resulting fluorescence colors depended on salt concentration, pointing to the complexity of controlled cluster synthesis on complex DNA scaffolds. The authors then incorporated Ag_*N*_-DNA-stabilizing hairpins into a hybridization chain reaction (HCR), with wire formation only after addition of an additional DNA strand (Fig. 22B). Ag_*N*_-DNAs have also been incorporated into DNA hydrogels.^221^

**Fig. 23:**
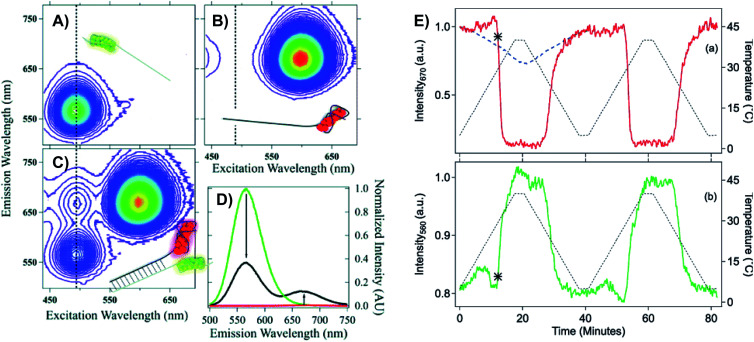
(A) Excitation–emission maps (EEMs) for an HPLC-purified green-emissive donor Ag_*N*_-DNA, (B) red-emissive acceptor Ag_*N*_-DNA, (C) and the WC-paired clamp. The EEM of the duplex is not simply an addition of (A) and (B) because FRET causes emission of the acceptor *via* excitation of the donor, evidenced by two peaks along the black dashed line in (C). (D) Emission spectra for 490 nm excitation of donor (green), acceptor (red), and WC pair (black). (E) FRET is cycled (intensity: green and red lines) by thermal melting (temperature: dashed black line) and reformation of the FRET pair.^222^ Adapted from Schultz, *et al.*, (ref. 222) with permission from the American Chemical Society. Copyright 2013.

Only two works have assembled purified Ag_*N*_-DNAs in order to approach atomic precision over cluster size in multi-cluster assemblies. Schultz, *et al.*, developed DNA “clamps” for dual-color Ag_*N*_-DNA pairs which exhibited Förster resonance energy transfer (FRET) between donor and acceptor Ag_*N*_-DNAs.^222^ DNA clamps were designed by appending complementary tails of A and T bases^13^ to templates for a green-emissive Ag_*N*_-DNA donor (Fig. 23A) and a red-emissive Ag_*N*_-DNA acceptor (Fig. 23B), which have a 6 nm Förster radius.^188^ After HPLC purification of individual Ag_*N*_-DNAs, various geometries of clamps were formed by WC pairing. For clamps where donor and acceptor were held within <6 nm, donor excitation produced acceptor emission (*e.g.*Fig. 23C), with >60% FRET efficiency estimated by donor quenching (Fig. 23D) and assuming no isolated donor is present^186^ (use of excess of acceptor increased the likelihood of all donors in the paired state). FRET could be repeatedly cycled by heating and cooling, corresponding to cyclic melting and reforming of the DNA clamp (Fig. 23E). The clamp design is somewhat general and was demonstrated with a different acceptor cluster. Notably, Schultz, *et al.*, found that HPLC purification was essential to observing FRET due to low chemical yield of Ag_*N*_-DNA synthesis; without purification, very few clamps contain both donor and acceptor clusters.^222^ Recently, Zhao, *et al.*, observed FRET between donor and acceptor Ag_*N*_-DNAs without prior purification by synthesizing Ag_*N*_-DNAs within surfactant reverse micelles with 5–10 nm diameters.^223^ By this method, a fraction of the micelles contained both donor and acceptor clusters confined together within a “nanocage” whose size is of the length scale of the Förster radius of the pair. This method also enabled spectroscopy-based measurement of micelle diameter in agreement with more laborious cryo-electron microscopy, suggesting that Ag_*N*_-DNA-based FRET may be a promising route to size measurement of biological “nanocage” structures.^223^

**Fig. 24:**
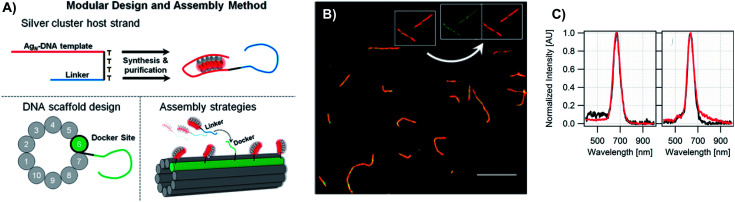
(A) Scheme of design method for modular Ag_*N*_-DNA template strand with linker, DNA nanotube scaffold with complementary docker strand, and assembly. (B) Spinning disc confocal microscopy of nanotubes labeled with 670 nm emissive Ag_*N*_-DNAs (red color) and FAM (organic dye, green) embedded in a polyvinyl alcohol film shows that Ag_*N*_-DNAs bind to the DNA nanotubes. Scale bar: 10 μm. (C) UV-excited fluorescence spectra of Ag_15_ (left) and Ag_14_ (right) free in solution (black) and after attachment to nanotubes with an excess of docker sites to ensure complete binding.^200^ Adapted from Copp, *et al.*, (ref. 200) with permission from the American Chemical Society. Copyright 2015.

Copp, *et al.* presented a modular design strategy for multifunctional DNA templates with distinct Ag_*N*_-DNA stabilizing regions and single-stranded “linker” regions.^200^ This strategy exploits large data libraries^57^ to identify 10-base DNA strands which do not foster fluorescent Ag_*N*_-DNA growth. These strands are candidate linkers to extend an Ag_*N*_-DNA template strand while leaving the cluster unchanged. Candidate linkers are appended to the DNA sequence of an HPLC-stable Ag_*N*_-DNA (Fig. 24A) and experimentally screened to determine if linkers leave Ag_*N*_-DNA optical spectra unshifted, signalling little to no change in cluster geometry. A complementary “docker” site is then engineered onto a DNA nanotube (Fig. 24A). Following HPLC purification of the Ag_*N*_-DNA, multi-cluster assembly occurs by WC pairing of linker and docker strands (Fig. 24B). Ag_*N*_-DNAs with atomically selected sizes are unperturbed after binding to the nanotubes, as supported by unchanged spectral shapes after assembly (Fig. 24C). Future studies are needed to confirm labelling efficiency. The method is general to multiple sizes of Ag_*N*_-DNAs and linker sequences^200^ and could generalize to many types of DNA scaffolds, for precise control over both cluster geometry and orientation.

Recently, Yourston, *et al.*, thoroughly studied Ag_*N*_-DNAs formed on RNA nanorings with DNA “arms.”^224^ Because *in situ* synthesis was used to decorate arms with Ag_*N*_-DNAs, it is uncertain how many Ag_*N*_ were harboured on a given nanoring. Interestingly, placement of the ssDNA region on which Ag_*N*_ presumably formed affected not only fluorescence spectra of the clusters, indicating variations in size/shape and possibly rigidity, but also time stability: clusters formed within the nanoring were much more time stable, perhaps due to enhanced protection from redox reactions which can blue-shift Ag_*N*_-DNA emission over time.^35,225^ Studies such as these will be important for assessing the practicality of DNA-based Ag_*N*_-DNA arrays as functional materials.

The heterogeneous mixture of products and low chemical yield of Ag_*N*_-DNA synthesis can prohibit precise Ag_*N*_-DNA arrays by direct synthesis onto a DNA nanostructure. Additionally, we pointed out in a previous section that one should also not *a priori* assume that the envisioned WC base pairing of the DNA nanostructure will be maintained once silver is introduced.

Assembly methods which instead rely on WC pairing after purification have their own limitations due to the limits of purity after HPLC and due to labelling efficiency of the DNA nanostructure by binding of Ag_*N*_-DNA linkers to each docker site; this may be overcome by adding an excess of Ag_*N*_-DNAs. Much more work is required to realize precise cluster arrays by either method.

## 7. Future directions and challenges

Significant recent progress has been made in understanding the structure–property relations of Ag_*N*_-DNAs and achieving their rational design. These advances were enabled by new experimental and computational strategies to purify and size Ag_*N*_-DNAs, to select new DNA templates for especially fluorescent Ag_*N*_-DNAs, and to crystallize Ag_*N*_-DNAs for structure determination, as discussed in this review. Here, we discuss outstanding challenges in this field and areas of especial promise, which we hope will catalyze new research directions in this important field.

### 7.1 Near-infrared emissive Ag_*N*_-DNAs

Nearly all reported Ag_*N*_-DNAs exhibit *λ*_em_ in the 500–750 nm range.^57^ The most well-studied NIR Ag_*N*_-DNAs have been developed by Petty and coauthors.^15,33,60,183,196^ High-throughput studies by Copp, *et al.*, uncovered additional NIR emissive clusters,^175^ and the Vosch group has characterized several of these recently discovered NIR Ag_*N*_-DNA, including one with an unusually large Stokes shift^176^ and one with an impressively high 73% quantum yield.^190^ These quantum yields are competitive with organic fluorophores, making Ag_*N*_-DNAs promising for development of biolabels in the NIR tissue transparency windows.^191^

**Fig. 25:**
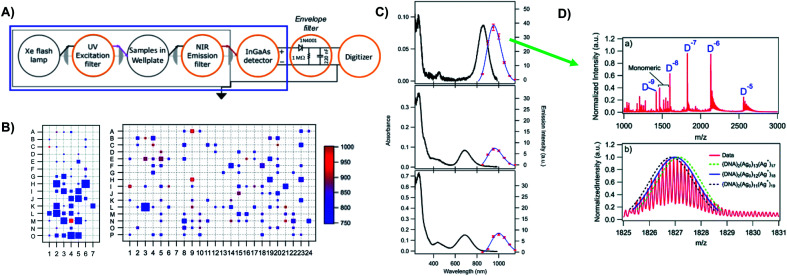
(A) Schematic of well plate reader for NIR rapid screening of candidate fluorophores,^226^ using an InGaAs PIN-type femtowatt photodetector. Adapted from Swasey, *et al.*, (ref. 226) with permission from AIP Publishing. (B) Colormaps of well plates containing Ag_*N*_-DNAs scanned using the modified plate reader, with box colors indicating peak emission wavelength and box size indicating relative fluorescence intensity.^23^ (C) Absorbance and emission spectra for three NIR-Ag_*N*_-DNAs identified in (B) and purified by HPLC, including the longest-wavelength emitting Ag_*N*_-DNA identified to date (bottom panel). (D) Mass spectrum of the Ag_*N*_-DNA associated with the top panel of (C), as measured using ESI-MS.^23^ (B–D) Adapted from Swasey, *et al.*, (ref. 23) with permission from the Royal Society of Chemistry.

Until recently, only two Ag_*N*_-DNAs with *λ*_em_ > 800 nm were reported,^196^ and it was assumed that NIR Ag_*N*_-DNAs are inherently rare compared to their visibly emissive counterparts. However, because Ag_*N*_-DNA studies employ UV-Vis optimized photodetectors commonly used for spectroscopy in the chemical and biological sciences, for which sensitivity is poor above ∼800 nm, it is possible that many NIR-emissive Ag_*N*_-DNAs have simply gone undetected. Swasey and Nicholson, *et al.*, developed a custom NIR well plate reader equipped with an InGaAs detector to search for NIR fluorophores in high-throughput (Fig. 25A).^226^ Using this tool to scan ∼750 Ag_*N*_-DNA samples, 161 previously unidentified NIR-emissive Ag_*N*_-DNAs were uncovered (Fig. 25B). This huge abundance of NIR products was unexpected because the scanned Ag_*N*_-DNAs were stabilized by randomly selected DNA template sequences^57^ or by oligomers previously designed for visible fluorescence.^175,215^ Among the newly discovered Ag_*N*_-DNAs were found the longest wavelength-emissive Ag_*N*_-DNA to date, with 999 nm peak fluorescence emission^23^ (Fig. 25C) and the largest Ag_*N*_-DNA to date, an Ag_30_ with 12 Ag^0^ and 18 Ag^+^ (Fig. 25D). A directed search for NIR Ag_*N*_-DNAs, using the informatics methods described in Section 5, is highly promising for discovery of new NIR Ag_*N*_-DNAs.

### 7.2 Ag_*N*_-DNA photophysics

Both experimental and computational efforts are needed to further our understanding of the fluorescence process in Ag_*N*_-DNAs, including the nature of the initial excited state, the relaxation process(es) leading to the origins of Stokes shifts for these emitters, and the roles of both the Ag_*N*_ and the surrounding nucleobases in governing excited state properties. While a zoo of Ag_*N*_ clusters stabilized by different ligands have been described in literature, their optical properties largely differ from the distinctive features of Ag_*N*_-DNAs described in Section 4. Zeolite stabilized Ag_*N*_ clusters display mainly strong UV absorption bands, with emissive excited-state decay stretching from picoseconds to the microsecond range.^227,228^ Similarly, the Mak group recently reported an intriguing octahedral silver cluster with 95% fluorescence quantum yield and microsecond-scale fluorescence decay times caused by thermally activated delayed fluorescence.^229^ Such microsecond-scale fluorescence decay times have not yet been observed for purified Ag_*N*_-DNAs. Only microseconds-lived dark states of Ag_*N*_-DNAs have been reported.

The unusual rod-like geometry of HPLC-stable Ag_*N*_-DNAs,^24^ which has been confirmed in recent crystal structures of NIR Ag_*N*_-DNAs,^25,68,69^ makes Ag_*N*_-DNAs particularly interesting experimental systems for the study of collective electronic excitations in molecular-like materials.^148,151–153^ With only *N*_0_ = 4–12 effective valence electrons in Ag_*N*_-DNAs characterized thus far by HR-MS,^23,24,57^ Ag_*N*_-DNAs lie well below the atomic size identified as the onset of plasmonic excitations in monolayer-protected gold clusters.^230^ However, the high aspect ratios of some identified Ag_*N*_-DNAs^147^ may make certain Ag_*N*_-DNAs better approximated as atomic silver rods, which computational studies have shown to exhibit plasmonic-like excitations.^136,139,144^ Future studies probing the ultrafast excited state dynamics of Ag_*N*_-DNAs are needed to better understand whether, or to what degree, collective electronic excitations are involved in the luminescence process of Ag_*N*_-DNAs.

Another feature of Ag_*N*_-DNA photophysics which remains poorly understood is the exact nature of the UV excitation process, which for the case of pure Ag_*N*_-DNA solutions leads to the same fluorescence spectral shapes as visible/NIR excitation (Fig. 1B).^21^ Due to DNA's complex and elegant excited state dynamics,^231^ it is possible that DNA imbues Ag_*N*_ with similar properties. Berkadin, *et al.*, have computationally examined the UV excitation process in Ag_*N*_-DNAs using molecular dynamics (MD) to simulate thread-like silver clusters in a DNA duplex, followed by DFT-based tight binding to calculate the electronic dynamics of the relaxed structure. Interesting, UV excitation results in a net negative charge transfer to the cluster, due to promotion of electrons from the localized π state of the DNA to the cluster.^232^ Such simulations performed on the recently reported crystal structures would be of great interest.^22,25,68,69^ Furthermore, recent experiments by the Kohler group on Ag^+^–nucleobase complexes are also promising for enhancing our understanding of this aspect of Ag_*N*_-DNA photophysics,^113,233^ with their very recent study finding evidence for an extremely long-lived, ∼10 ns excited state in a C_20_–Ag^+^-C_20_ duplex.

### 7.3 Rational sensor design

Many chemical and biomolecular sensing schemes employing Ag_*N*_-DNAs have been developed, such as NanoCluster Beacons,^32,39^ ratiometric sensors,^234–236^ and microRNA sensors^14,119^ (more complete list in a past review^74^). Designing these sensors is extremely challenging, and designs may not generalize because silver clusters are not confined only to the expected regions of a probe.^234^ Further, the mechanisms underlying the function of these sensors remain uncertain in most cases, although color and brightness changes are likely due to restructuring of Ag_*N*_-DNAs.^34,62,138^ Recent efforts have focused on strategies to improve sensitivity and selectivity of Ag_*N*_-DNA sensors, such as by addressing the low chemical yield of these clusters.^237^ Due to the complexity of designing Ag_*N*_-DNA sensors, we propose that high-throughput experimentation combined with machine learning approaches may be a useful path forward. The Yeh group has recently pioneered high-throughput screening of Ag_*N*_-DNA sensors, which may significantly expedite progress in this area.^238–240^

Purified Ag_*N*_-DNAs could also serve as sensitive nanophotonic sensors. The photophysical properties of pure Ag_*N*_-DNAs have also been shown to exhibit sensitivity to temperature,^176,185^ refractive index,^147,189^ and viscosity.^176^ Combined with advancement in the ability to pattern DNA nanostructures with Ag_*N*_-DNAs, it may be possible to design sensors which colocalize Ag_*N*_-DNAs with analyses of interest for nanoscale measurements.

### 7.4 Non-natural polynucleic acids as cluster templates

While DNA has been well-studied as a template for silver clusters, and RNA to a lesser extent,^46^ much less is known about the suitability of non-natural polynucleic acids to template silver clusters.^241^ Because RNA is less flexible than DNA, it has been noted that RNA may be less suitable as a scaffold for Ag_*N*_ if significant flexibility of oligomer ligands is required for a given cluster geometry.^69^ Synthetic polynucleic acids could expand cluster structures and geometries, enhance stabilities, and imbue added functionalities. In addition to the four natural nucleobases, numerous artificial nucleobases have well-studied affinity for silver and other metals.^77–80^ A large range of fluorescent nucleotide analogues are currently available, with more being actively developed continuously.^242–244^ These bases could shift the universal UV excitation peak into the blue region of the visible spectrum, and FRET experiments could help elucidate the energy transfer processes in Ag_*N*_-DNAs and even unravel distances and proximity of selected nucleobases to the Ag_*N*_ cluster. Also of interest are chemical modifications developed for therapeutics to reduce enzymatic nucleotide digestion,^245^ which have been reported to template Ag_*N*_-DNAs,^246^ and other backbone modifications which would influence ligand conformation and, therefore, possible stabilized cluster geometries. Future studies are needed in this promising area.

### 7.5 Uncharacterized toxicity and biocompatibility of Ag_*N*_-DNAs

Ag_*N*_-DNAs are often touted as nontoxic and biocompatible fluorophores,^64,65,70–74^ but very few studies have established these properties.^247,248^ While Ag^+^ is certainly less toxic than other heavy metals that compose luminescent nanoparticles such as quantum dots,^249^ it is also a common environmental metal pollutant.^250^ Ag nanoparticles can break down in the body in a range of different ways, resulting in toxicities due to either the nanoparticles themselves or to Ag^+^ and silver salts. Ag nanoparticles are also finding use as anti-cancer therapeutics,^251^ adding further complexity to our understanding of the toxicity of Ag_*N*_-DNAs. In-depth studies of the toxicities of Ag_*N*_-DNAs and their uptake and possible clearing from tissues and organisms are needed to advance their applications in the biomedical sciences and to ensure environmentally responsible use and disposal.

### 7.6 Enhancing stability

While Ag_*N*_-DNAs are often touted as extremely photostable and/or chemically stable, degradation of Ag_*N*_-DNAs in biologically relevant solutions and in the presence of living cells is a significant hindrance to their practical use in bioimaging.^252^ To overcome this challenge, Jeon, *et al.*, encapsulated Ag_*N*_-DNAs within silica nanoparticles, significantly increasing cluster chemical stability.^253^ The encapsulated Ag_*N*_-DNAs can also be used to monitor the stability of their silica nanoparticle hosts in various biological media.^254^*In situ* synthesis of Ag_*N*_-DNAs within DNA hydrogels can improve photostability, likely by shielding clusters from oxidative degradation.^255^ Lyu, *et al.*, reported significantly enhanced chemical stability and increased cellular uptake of Ag_*N*_-DNAs modified by cationic polyelectrolytes.^252^

## 8. Conclusions

Ag_*N*_-DNAs lie at the unique intersection of metal cluster science and DNA nanotechnology, combining the atomic precision of ligand-stabilized metal clusters with the sequence programmability of DNA nanomaterials. Their photophysical properties also provide a window into the regime between behavior associated with single small molecules and behavior associated with nanoparticles. For these reasons, the study and engineering of Ag_*N*_-DNAs are both extremely challenging and extremely promising. Here, we have reviewed recent advances in the fundamental understanding of these nanoclusters, with a focus on studies of purified Ag_*N*_-DNAs with chromatographically selected sizes. The latest (and evolving) findings of Ag_*N*_-DNA structure and the nature of the DNA–silver interaction have been discussed. Photophysical studies, particularly of purified Ag_*N*_-DNAs, have been summarized. The current understanding of how DNA sequence selects for cluster size and optical properties has been reviewed, as have emerging methods for predictive design of Ag_*N*_-DNAs and their larger organization into multi-cluster arrays. We provide perspectives on emerging areas of interest and significant unanswered questions related to these fluorescent clusters in the hopes of stimulating researchers to explore these fascinating nanomaterials.

## Conflicts of interest

There are no conflicts to declare.

## Supplementary Material
